# Evaluation of novel factor Xa inhibitors from *Oxya chinensis sinuosa* with anti-platelet aggregation activity

**DOI:** 10.1038/s41598-017-08330-1

**Published:** 2017-08-11

**Authors:** Wonhwa Lee, HeeSeung Lee, Mi-Ae Kim, Joonhyeok Choi, Kyung-Min Kim, Jae Sam Hwang, MinKyun Na, Jong-Sup Bae

**Affiliations:** 1 College of Pharmacy, CMRI, Research Institute of Pharmaceutical Sciences, BK21 Plus KNU Multi-Omics based Creative Drug Research Team, Kyungpook National University, Daegu, 41566 Republic of Korea; 2 College of Pharmacy, Chungnam National University, Daejeon, 34134 Republic of Korea; 3 Department of Agricultural Biology, The National Academy of Agricultural Science, RDA, Wanju-gun, 55365 Republic of Korea; 4 Division of Plant Biosciences, School of Applied BioSciences, College of Agriculture and Life Science, Kyungpook National University, Daegu, 41566 Republic of Korea

## Abstract

The edible grasshopper *Oxya chinensis sinuosa* is consumed worldwide for its various medicinal effects. The purpose of this study was to investigate potential bioactive antithrombotic and antiplatelet compounds from *O. chinensis sinuosa*. Five *N*-acetyldopamine dimers (**1**–**5**) were isolated from *O. chinensis sinuosa* and compounds **1** and **2** were identified as new chemicals with chiral centers at H-2 and H-3 of the benzo-1,4-dioxane structure. Compounds **1**–**4** were found to have both FXa and platelet aggregation inhibitory activities. These compounds inhibited the catalytic activity of FXa toward its synthetic substrate, S-2222, by noncompetitive inhibition, and inhibited platelet aggregation induced by ADP and U46619. Furthermore, compounds 1–4 showed enhanced antithrombotic effects, which were assessed using *in vivo* models of pulmonary embolism and arterial thrombosis. The isolated compounds also showed anticoagulant effects in mice. However, compounds 1–4 did not prolong bleeding time in mice, as shown by tail clipping. *N*-Acetyldopamine dimers, including two new stereoisomers **1** and **2**, are novel antithrombotic compounds showing both FXa inhibition and antiplatelet aggregation activity with a low bleeding risk. Collectively, these results suggest that compounds 1–4 could serve as candidates and provide scaffolds for development of new antithrombotic drugs.

## Introduction

Globally, thrombosis plays a leading role in causing death and a major role in the onset of many cardiovascular diseases, such as acute coronary syndrome, myocardial infarction, and deep vein thrombosis^1^. Thrombus due to large anomalous coagulation can often cause reduced blood flow or ischemia in arteries or veins^1^. In response to blood vessel injuries, the blood coagulation cascade is initiated by the activation of zymogens, in which thrombin and activated coagulation factor X (FXa) play a key role. The prothrombinase complex consists of the serine protein, FXa, and the protein cofactor, Factor Va; FXa converts prothrombin to thrombin to link the intrinsic and extrinsic pathways^2^. Most thromboembolisms require anticoagulant therapy^1^. The unsatisfactory antithrombotic efficacy in clinical trials can explain vascular relapses, and it justifies current research to develop specific and potent antithrombotic agents. Therefore, studies to find new bioactive compounds with different mechanisms of action, improved efficacy, and no toxicity are needed^1^.

The early focus of research to find antithrombotic drugs was to develop thrombin inhibitors, attributable to its central role in thrombosis^3^. However, clinical studies have shown that the continuous production of thrombin from prothrombin is not blocked by direct inhibitors of thrombin^4, 5^. It is necessary to use high concentrations of thrombin inhibitors to achieve antithrombotic efficacy *in vivo*, which may lead to undesirable consequences of anticoagulation and ultimately increase the complications of hemorrhage. Thus, direct thrombin inhibitors have a narrow therapeutic range. In view of the bleeding risk, it may be safer to selectively inhibit blood clotting factors located upstream of thrombin. In this regard, FXa has recently gained attention as a target of new antithrombotic agents^6, 7^. Inhibiting FXa could attenuate the subsequent production of thrombin while maintaining a basal amount of thrombin for primary hemostasis^7^. In addition, selective inhibition of FXa has been shown to be superior to the application of heparin or direct thrombin inhibitors^8^.

Insects comprise the largest number of creatures on earth, more than 80%. Globally, 950,000 species of insects have been reported^9^. It has been estimated that more than 1,900 kinds of insects have been utilized as foods^10^. Since 2003, the Food and Agriculture Organization (FAO) has been conducting studies on edible insects in many countries. The most commonly consumed edible insects in the world belong to the orders Coleoptera (31%), Lepidoptera (18%), Hymenoptera (14%), Orthoptera (13%), Hemiptera (10%), and others (14%)^10^. Insects are recognized as an important food and medicinal resource. For example, many edible insects provide sufficient amounts of energy and protein, and meet amino acid requirements for humans^11^. The venom extracted from *Pachycondyla sennaarensis* (Hymenoptera) displayed protective activity against CCl_4_-induced nephrotoxicity^12^. Carmine and resilin were isolated from Diaspididae (Hemiptera) species and have been used as coloring additives and rubber-like biomaterials, respectively^13^. Honey from Hymenoptera has been utilized in the treatment of several skin disorders (e.g., bacterial infections, seborrheic dermatitis, and dandruff), and melon bug (*Coridius viduatus*, Hemiptera) oil is used to treat skin lesions in medicine^14, 15^.

*Oxya chinensis sinuosa* Mistshenko, an edible grasshopper, belongs to the phylum Arthropoda (Order: 54 Orthoptera, Family: Acrididae, subfamily: Oxyinae)^16^. Traditionally in Korea, it is known as the “famine relief insect” and has been used to treat cough, whooping cough, asthma, bronchitis, paralysis, and seizures^17^. Recently, the rice field grasshopper has been registered as a food in the Korean Food Standards Codex, Korea Food & Drug Administration (KFDA). Although the grasshopper, *O. chinensis sinuosa*, has long been used as food in Asia, there is little information on its chemical constituents or their activities, including antithrombotic and antiplatelet effects. During a screening for natural antithrombotic and antiplatelet products from insects, the ethyl acetate-soluble fraction derived from *O. chinensis sinuosa* showed potent inhibition of FXa generation in human umbilical vein endothelial cells (HUVECs). Herein, we describe for the first time the isolation of five *N*-acetyldopamine dimers, including two novel ones (**1** and **2**), from *O. chinensis sinuosa* and investigation of their antithrombotic and antiplatelet functions. The antithrombotic activity of the compounds is further characterized in animal models. To the best of our knowledge, this is the first report regarding the antithrombotic and antiplatelet effects of *O. chinensis sinuosa*, and the discovery of new compounds demonstrating dual inhibition of FXa and platelet aggregation.

## Results

### Structure Determination of *N*-Acetyldopamine Dimers from *O. chinensis sinuosa*

**Figure 1:**
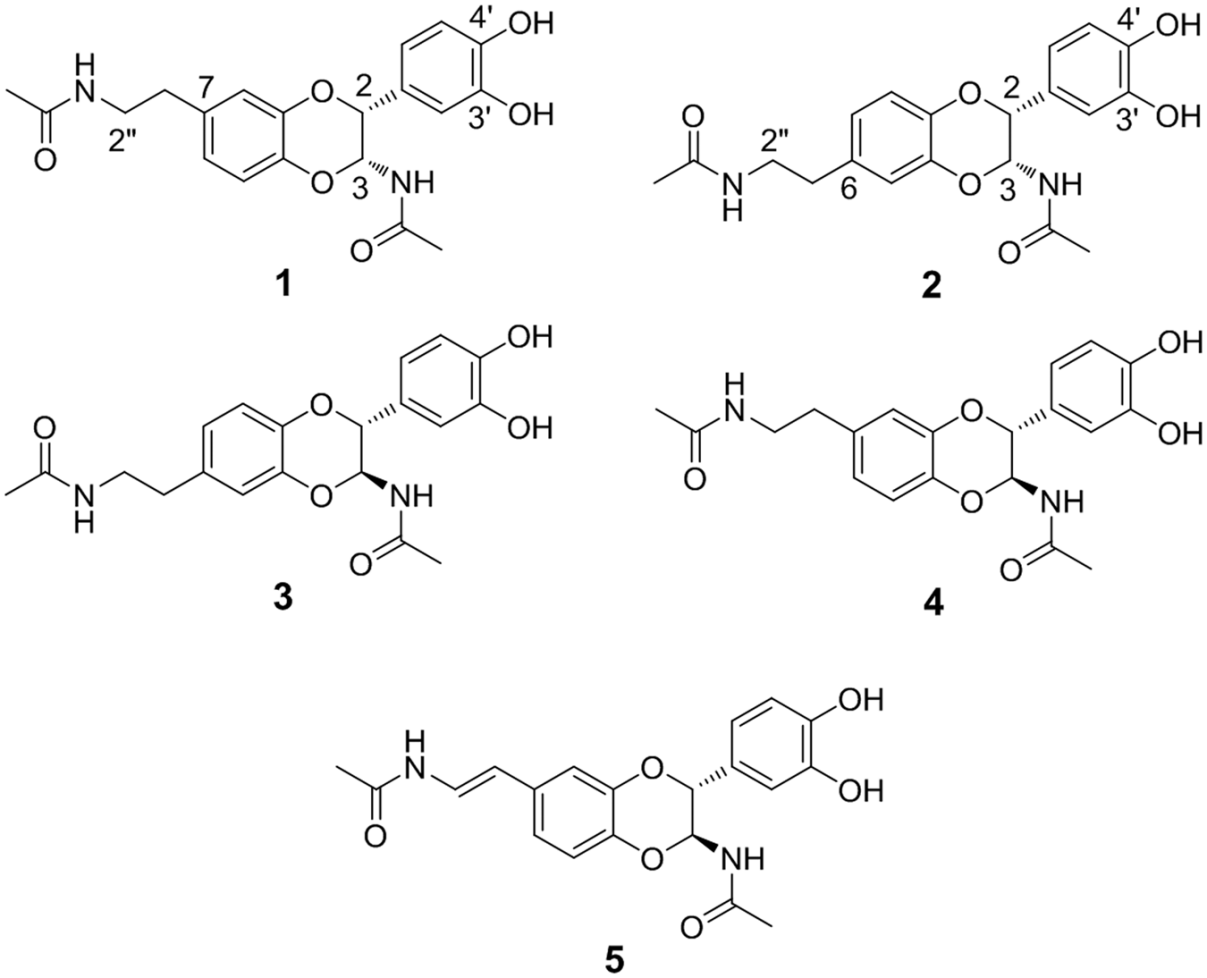
Structure of active compounds 1–5 isolated from *O. chinensis sinuosa*.

An ethanol extract of the insect, *O. chinensis sinuosa*, was subjected to several chromatographic techniques to afford five *N*-acetyldopamine dimmers (Fig. 1). Two new compounds (**1** and **2**) were identified based on analysis of spectroscopic data (i.e., high-resolution electrospray ionization mass spectrometry (HRESIMS), circular dichroism, optical rotation, 1D and 2D NMR data).

**Figure 2:**
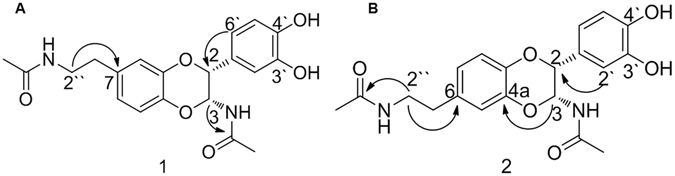
Key HMBC () correlations of compounds 1 and 2.

**Figure 3:**
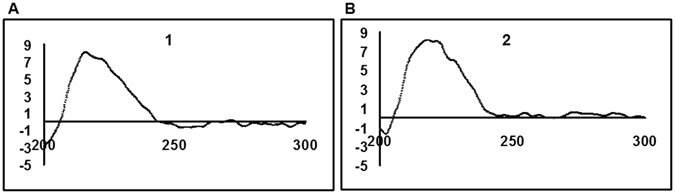
The CD spectra of compounds 1 and 2.

Compound **1**, ${[{\rm{\alpha }}]}_{D}^{23}$ - 21.5 (MeOH, *c* 0.1), was isolated as a yellowish, amorphous powder. The molecular formula was established as C_20_H_22_N_2_O_6_ based on HRESIMS (*m/z* 409.1376 [M + Na]^+^, calcd. for C_20_H_22_N_2_O_6_Na 409.1346) and ^13^C-NMR data. The ^1^H-NMR (Table 1) and ^13^C-NMR (Table 2) data were typical of those of dopamine derivatives^18^. The ^1^H-NMR data (Table 1) showed two ABX-type spin systems in the aromatic region (*δ*_H_ 6.83~6.78, overlap), two methylenes (*δ*_H_ 3.35, t, *J* = 3.6 Hz and 2.71, t, *J* = 7.2 Hz), and two methines (*δ*_H_ 6.03, d, *J* = 1.7 Hz and 5.11, d, *J* = 1.5 Hz), with four additional signals due to OH and NH protons. The ^13^C-NMR data exhibited twelve signals in the aromatic region in addition to signals for two carbonyl groups (*δ*_C_ 174.1, 173.3). The heteronuclear multiple bond correlation (HMBC) cross peaks between H-2″ (*δ*_H_ 3.35, t, *J* = 3.6 Hz) and C-7 (*δ*_C_ 134.2) suggested that the N-acetylamino-2-ethyl group is positioned at C-7 (Fig. 2). The observed HMBC correlations between H-3 (*δ*_H_ 6.03, d, *J* = 1.7 Hz) and the carbonyl group (*δ*_C_ 174.1) suggest that another *N*-acetylamide group is connected to the C-3 position (Fig. 2). The 3′,4′-dihydroxybenzene ring was confirmed to be located at the C-2 position by HMBC cross peaks between H-2′/H-6′ (*δ*_H_ 6.83~6.78, overlap) and C-2 (*δ*_C_ 77.2). The stereochemistry at H-2 and H-3 of the 1,4-dioxane ring was different from that previously reported^18^. The small coupling constants of H-2 and H-3 (*J*_H-2/H-3_ = 1.6 Hz) were distinguished from those of 2,3-*trans*-*N*-acetyldopamine dimer (*J*_H-2/H-3_ = 7.3 Hz)^18^, indicating the *cis* configuration between H-2 and H-3 of compound **1**. The absolute configuration of H-2 and H-3 was then determined by CD spectroscopic data analysis, where the positive Cotton effect at 235 nm indicated the 2 *R* configuration of compound **1** (Fig. 3)^19^. QCollectively, the structure of compound **1** was established as (2 *R*,3 *R*)-2-(3′,4′-dihydroxyphenyl)-3-acetylamino-7-(*N*-acetyl-2″-aminoethyl)-1,4-benzodioxane, and was named oxyamide A.

**Table 1:** 1H NMR Spectroscopic Data (600 MHz, methanol-*d*_4_) for Compounds **1–5**.

position	**1**	**2**	**3**	**4**	**5**
	*δ*_H_ (*J* in Hz)	*δ*_H_ (*J* in Hz)	*δ*_H_ (*J* in Hz)	*δ*_H_ (*J* in Hz)	*δ*_H_ (*J* in Hz)
2	5.11, d (1.5)	5.09, d (1.0)	4.68, d (7.2)	4.69, d (7.1)	4.71, d (7.1)
3	6.03, d (1.7)	6.03, d (1.3)	5.68, d (7.2)	5.67, d (7.1)	5.69, d (7.2)
5	overlap	overlap	overlap	overlap	6.76, d (8.1)
6	overlap			overlap	6.86, dd (1.9, 8.4)
7		overlap	overlap		
8	overlap	overlap	overlap	overlap	6.81, d (8.4)
4a					
8a					
1′					
2′	overlap	overlap	overlap	overlap	6.90, d (1.8)
3′					
4′					
5′	Overlap	Overlap	overlap	overlap	6.85, brs
6′	Overlap	Overlap	overlap	overlap	6.74, dd (1.6, 8.1)
1″	2.71, t (7.2)	2.70, t (7.1)	2.69, t (7.3)	2.69, t (7.2)	6.10, d (14.7)
2″	3.35, t (3.6)	3.35, t (5.5)	3.35, m	3.35, t, (7.1)	7.30, d (14.7)
CH_3_	1.92, s	1.90, s	1.91, s	1.90, s	2.03, s
	1.84, s	1.84, s	1.87, s	1.87, s	1.87, s

**Table 2:** 13C NMR Spectroscopic Data (150 MHz, methanol-*d*_4_) for Compounds **1–5**.

position	**1**	**2**	**3**	**4**	**5**
	*δ*_C_, type	*δ*_C_, type	*δ*_C_, type	*δ*_C_, type	*δ*_C_, type
2	77.2, CH	77.1, CH	78.2, CH	78.3, CH	78.4, CH
3	77.0, CH	77.2, CH	78.3, CH	78.3, CH	78.3, CH
5	118.5, CH	118.7, CH	118.2, CH	117.9, CH	116.1, CH
6	123.7, CH	134.8, C	134.3, C	123.2, CH	120.3, CH
7	134.2, C	123.1, CH	123.1, CH	134.2, C	131.9, C
8	118.7, CH	118.5, CH	117.9, CH	118.1, CH	118.3, CH
4a	140.9, C	143.3, C	143.5, C	142.2, C	142.6, C
8a	144.6, C	142.2, C	143, C	144.3, C	144.6, C
1′	128.2, C	128.2, C	128.8, C	128.8, C	128.7, C
2′	114.5, CH	114.5, CH	115.6, CH	115.6, CH	114.7, CH
3′	146.6, C	146.6, C	146.5, C	146.5, C	146.5, C
4′	146.7, C	146.8, C	147.2, C	147.1, C	147.2, C
5′	116.2, CH	116.2, CH	116.1, CH	116.1, CH	115.6,, CH
6′	118.8, CH	118.8, CH	120.6, CH	120.6, CH	120.6,, CH
1″	35.8, CH_2_	35.8, CH_2_	35.8, CH_2_	35.8, CH_2_	114, C
2″	42.2, CH_2_	42.2, CH_2_	42.1, CH_2_	42.1, CH_2_	122.9, C
CH_3_	22.6, CH_3_	22.5, CH_3_	22.6, CH_3_	22.6, CH_3_	22.6, CH_3_
	22.3, CH_3_	22.3, CH_3_	22.5, CH_3_	22.5, CH_3_	22.6, CH_3_
CO	174.1, C	174.1, C	173.3, C	173.3, C	173.2, C
	173.3, C	173.2, C	173.2, C	173.3, C	170.6, C

Compound **2** ${[{\rm{\alpha }}]}_{D}^{23}$ - 42 (MeOH, *c* 0.1) was isolated as a yellowish, amorphous powder. The molecular formula was established as C_20_H_22_N_2_O_6_ from the HRESIMS data exhibiting the sodium adduct at *m/z* 409.1376 (calcd. for C_20_H_22_N_2_O_6_Na 409.1345) and the ^13^C-NMR data (Table 2). The ^1^H-NMR (Table 1) and ^13^C-NMR (Table 2) spectroscopic data of compound **2** were similar to those of compound **1**, except for the position of the *N*-acetylamino-2-ethyl moiety. The HMBC cross peaks between H-2″ (*δ*_H_ 3.35, t, *J* = 5.5 Hz) and C-6 (*δ*_C_ 134.8) indicated that the *N*-acetylamino-2-ethyl group should be connected to C-6 (Fig. 2). The HMBC cross peaks between H-3 (*δ*_H_ 6.03, d, *J* = 1.3 Hz) and C-4a (*δ*_C_ 143.3) confirmed the *cis* configuration between H-2 and H-3, while the HMBC correlations for *trans* isomers were not observed^18^. The relative configuration of H-2 and H-3 was determined to be *cis*, which was deduced from the small coupling constant value (*J*_H-2/H-3_ = 1.2 Hz) in the ^1^H-NMR data. The absolute configuration of the H-2 and H-3 positions was established to be 2 *R* based on CD spectroscopic data (positive Cotton effect at 235 nm) (Fig. 3). Thus, the structure of compound **2** was established as (2 *R*,3 *R*)-2-(3′,4′-dihydroxyphenyl)-3-acetylamino-6-(*N*-acetyl-2″-aminoethyl)-1,4-benzodioxane, and was named oxyamide B.

Compound **3** ${[{\rm{\alpha }}]}_{D}^{23}$ - 38.2 (MeOH, *c* 0.1) was isolated as a yellowish, amorphous powder. The molecular weight was acquired from the electrospray ionization mass spectrometry (ESIMS) proton adduct ion at *m/z* 387 (calcd. [M + H]^+^, *m/z* 387). Inspection of the CD and NMR data revealed that the structure of compound **3** should be an *N*-acetyldopamine dimer. Considering the coupling constants between H-2 and H-3 (*J*_H-2/H-3_ = 7.2 Hz), the relative configuration of H-2 and H-3 was confirmed as the *trans*-form. The structure of compound **3** was finally identified as (2 *R*,3 *S*)-2-(3′,4′-dihydroxyphenyl)-3-acetylamino-6-(*N*-acetyl-2″-aminoethyl)-1,4-benzodioxane^18^.

The ESIMS data of compound **4** ${[{\rm{\alpha }}]}_{D}^{23}$ - 36.2 (MeOH, *c* 0.1), purified as a yellowish and amorphous powder, showed a molecular ion signal at *m/z* 387 (calcd [M + H]^+^, *m/z* 387). The NMR and CD spectroscopic data of compound **4** resembled that of compound **1**. The chemical shifts of H-2 (*δ*_H_ 4.69, d, *J* = 7.1 Hz) and H-3 (*δ*_H_ 5.67, d, *J* = 7.1 Hz) were different from those of compound **1**. Furthermore, the coupling constant value (*J*_H-2/H-3_ = 7.1 Hz) observed in the ^1^H-NMR data of compound **4** indicated that the relative configuration of H-2 and H-3 should be *trans*. Consequently, the structure of compound **4** was proposed as (2 *R*,3 *S*)-2-(3′,4′-dihydroxyphenyl)-3-acetylamino-7-(*N*-acetyl-2″-aminoethyl)-1,4-benzodioxane^18^.

Compound **5** ${[{\rm{\alpha }}]}_{D}^{23}$ - 31.4 (MeOH, *c* 0.1) was isolated as a yellowish, amorphous powder. A difference of 2 mass units (*m/z* 407 [M + Na]^+^) compared to compounds **1**-**4** (*m/z* 409 [M + Na]^+^ for C_20_H_22_N_2_O_6_Na) suggested the presence of an additional double bond in compound **5**. The ^1^H-NMR data of compound **5** were similar to those of compound **4**, except for olefinic signals for H-2″ (*δ*_H_ 7.30, d, *J* = 14.7 Hz) and H-1″ (*δ*_H_ 6.10, d, *J* = 14.7 Hz). Consequently, the structure of compound **5** was determined to be (2 *R*,3 *S*)-2-(3′,4′-dihydroxyphenyl)-3-acetylamino-7-(*N*-acetyl-2″-aminethylene)-1,4-benzodioxane^20^.

### Enzyme Inhibitory Effect of Isolated Compounds against Different Human Enzymes

Final concentrations of enzymes and substrates used in K_i_ determinations are shown in Table 3. The results of a chromogenic substrate assay demonstrate that compounds **1**–**4** are a potent inhibitor of human FXa with a *K*_i_ = 2.96, 4.13, 4.45, and 4.72 μM, respectively (Table 4). The selectivity of compounds **1**–**4** for FXa compared with a selection of other human enzymes was shown in Table 4. Compounds **1**–**4** are highly selective for FXa and demonstrates a selectivity ratio (based on the respective K_i_ values) of >10,000 for FXa versus thrombin, trypsin, elastase, plasmin, protein Ca, streptokinase, tPA, and urokinase.

**Table 3:** Final concentrations of enzymes and substrates used in Ki determinations.

Enzyme	Concentrations	Substrate
Factor Xa	1.8 mM	S-2765
α-Thrombin	35,280 μM	S-2238
Trypsin	0.32 mM	S-2222
Elastase	0.54 mM	L-1335
Plasmin	1.2 mM	S-2251
Protein Ca	3.2 mM	S-2366
Streptokinase	1.4 mM	S-2251
tPA	1.4 mM	S-2228
Urokinase	2.4 mM	S-2444

**Table 4:** Enzyme kinetics and selectivity of isolated compounds against different human enzymes.

Enzyme	Compound 1		Compound 2		Compound 3		Compound 4		Compound 5	
	K_i_^a^	Ratio^b^	K_i_	Ratio	K_i_	Ratio	K_i_	Ratio	K_i_	Ratio
Factor Xa	2.96 ± 0.25	1	4.13 ± 0.43	1	4.45 ± 0.37	1	4.72 ± 0.31	1	>250	n.d.
α-Thrombin	>300	>100	>500	>100	>500	>100	>500	>100	>250	n.d.
Trypsin	>300	>100	>500	>100	>500	>100	>500	>100	>250	n.d.
Elastase	>300	>100	>500	>100	>500	>100	>500	>100	>250	n.d.
Plasmin	>300	>100	>500	>100	>500	>100	>500	>100	>250	n.d.
Protein Ca	>300	>100	>500	>100	>500	>100	>500	>100	>250	n.d.
Streptokinase	>300	>100	>500	>100	>500	>100	>500	>100	>250	n.d.
tPA	>300	>100	>500	>100	>500	>100	>500	>100	>250	n.d.
Urokinase	>300	>100	>500	>100	>500	>100	>500	>100	>250	n.d.

^a^K_i_ is represented by the mean ± SD (n = 5), μM.

^b^Ratio = K_i_ enzyme/K_i_ Factor Xa.

n.d., not determined.

### Effect of the Isolated Compounds on Clotting Time *Ex Vivo* and *In Vivo*

**Figure 4:**
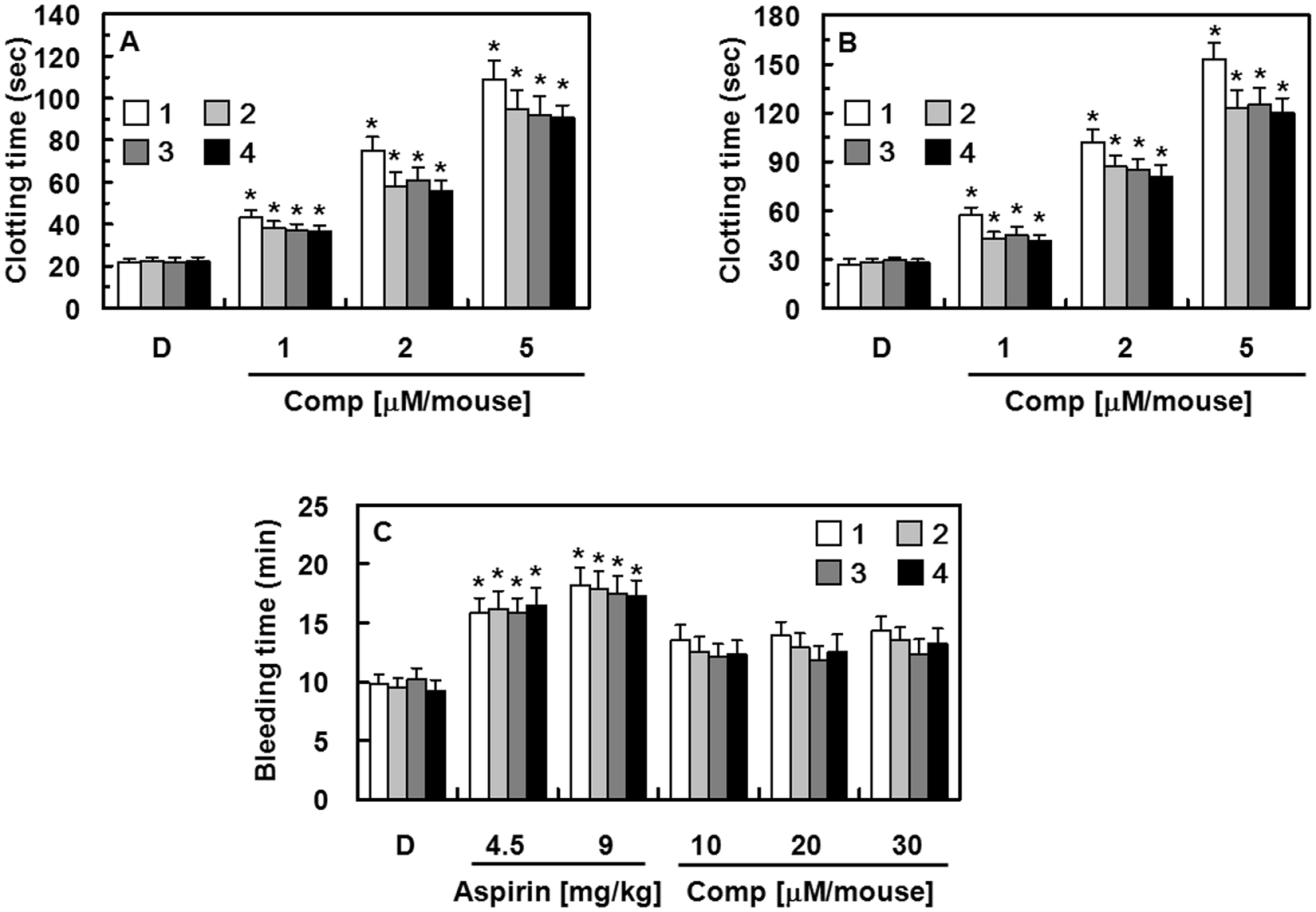
Effects of compounds 1–4 on clotting and bleeding time. (**A**) One hour after administration (intravenous injection) of compound **1** (white box), **2** (light gray box), **3** (dark gray box), or **4** (black box), blood was collected from the mice and platelet-poor plasma (PPP) was obtained by centrifugation at 2,000 × *g* for 10 min at room temperature to test *ex vivo* activated partial thromboplastin time (aPTT). (**B**) Each group received a daily intravenous injection of the indicated compound for four consecutive days. Fifteen minutes after the last administration, blood samples were collected and *in vivo* aPTT was measured. (**C**) Fifteen minutes after administration of each compound or aspirin (4.5 or 9 mg/kg for 30 min) or the vehicle, tail tips (3 mm long) were cut from each mouse, and the remaining tail was immediately immersed into saline at 37 °C. Accumulated bleeding times (including periods of re-bleeding) were recorded. D = 0.2% DMSO used as the vehicle control. Data are presented as means ± SD of three independent experiments. *p < 0.05 vs. vehicle alone, analyzed by one-way ANOVA, followed by Tukey multiple comparison testing.

To evaluate the effects of compounds **1**–**5** on coagulation parameters *ex vivo*, we measured the influence of the isolated compounds on activated partial thromboplastin time (aPTT) and prothrombin time (PT). Pre-administration of compounds **1**–**4** intravenously to mice significantly increased aPTT in a dose-dependent manner at doses ranging from 38 to 193 µg/kg (compounds **1**–**4**, Fig. 4A). However, compound **5** did not affect these measures (data not shown). The average circulating blood volume for mice is 72 mL/kg^21^. Because the average weight of mice used in this study was 27 g and the average blood volume was 2 mL, the dose of compounds **1**–**5** (38.6, 77.2, or 193.1 µg/kg) equaled a peripheral blood concentration of approximately 1, 2, and 5 µM, respectively. However, PT was not significantly higher in mice treated with compounds **1**–**5** than that of mice treated with vehicle only (data not shown). Compounds **1**–4 significantly prolonged blood clotting time in a dose-dependent manner as shown in the *in vivo* clotting time experiments. These data indicate that compounds **1**–**4** but not compound **5** have significant, dose-dependent anticoagulant effects *in vivo* (Fig. 4B).

### Effect of Compounds 1–5 on Platelet Aggregation

**Figure 5:**
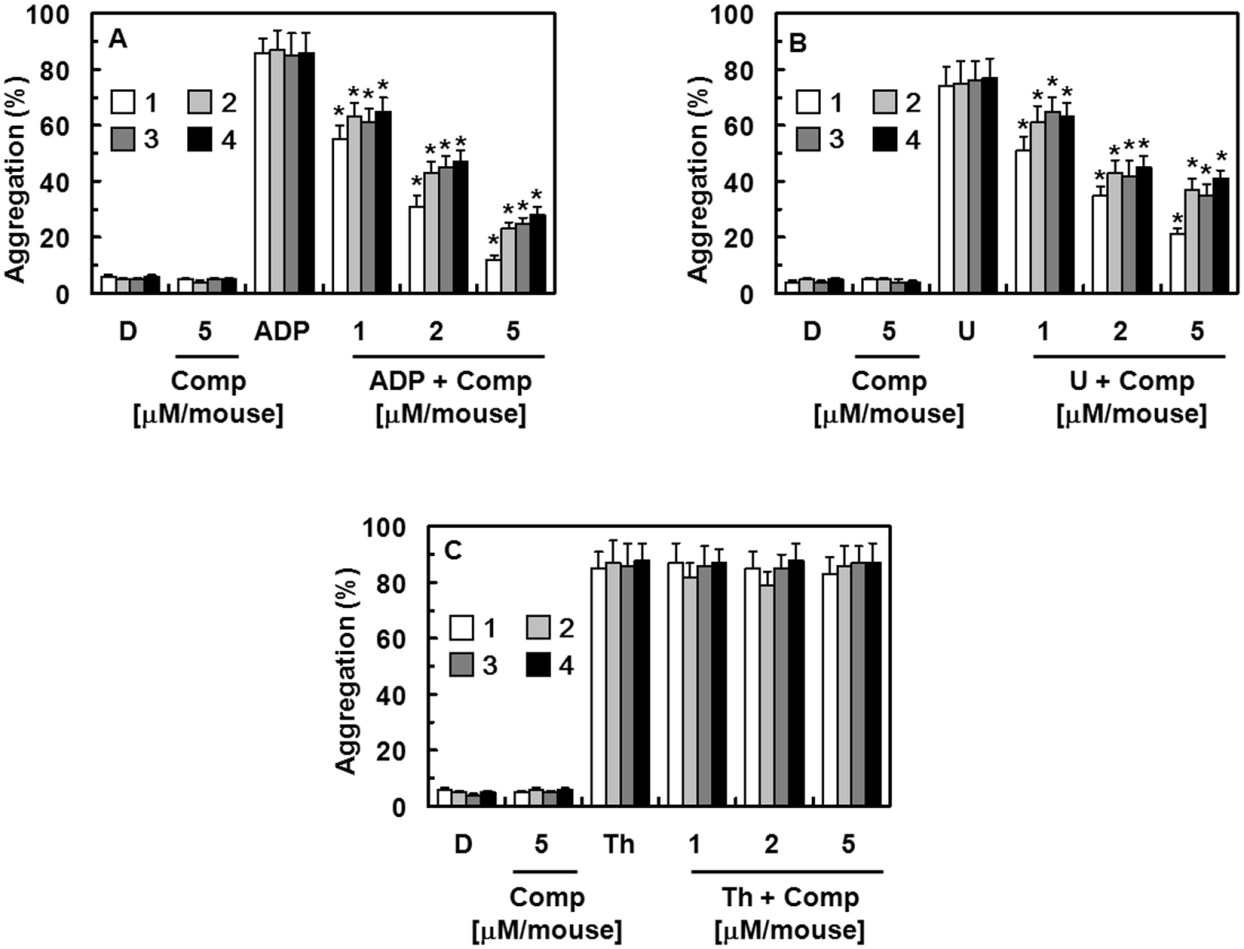
Effects of compounds 1–4 on agonist-induced platelet aggregation *in vitro*. Platelet-rich plasma **(**PRP) was preincubated for 5 min with different concentrations of compounds **1**–**4**, or vehicle. Platelet aggregation was initiated with ADP (A, 10 µM), U46619 (B, 6 µM), or thrombin (C, 3 U/mL). D = 0.2% DMSO used as the vehicle control. Data are presented as means ± SD of three independent experiments. ^*^ *p* < 0.05 vs. vehicle alone, analyzed by one-way ANOVA, followed by Tukey multiple comparison testing.

To study the effects of compounds **1**–**5** on platelet aggregation, we used mouse platelet-rich plasma (PRP) induced with various agonists as an *in vitro* model. The results show that compounds **1**–**4** selectively inhibit platelet aggregation induced by different agonists. As shown in Fig. 5, compounds **1**–**4** inhibited platelet aggregation induced by ADP (10 µM, Fig. 5A) and U46619 (a stable thromboxane A2 analogue/aggregation agonist, 6 µM, Fig. 5B) in a dose-dependent manner. However, the compounds did not inhibit thrombin-induced platelet aggregation (Fig. 5C). In addition, compound **5** did not affect the aggregation of platelets, regardless of induction (data not shown).

### Effects of Compounds 1–4 on FXa Activity and Production

**Figure 6:**
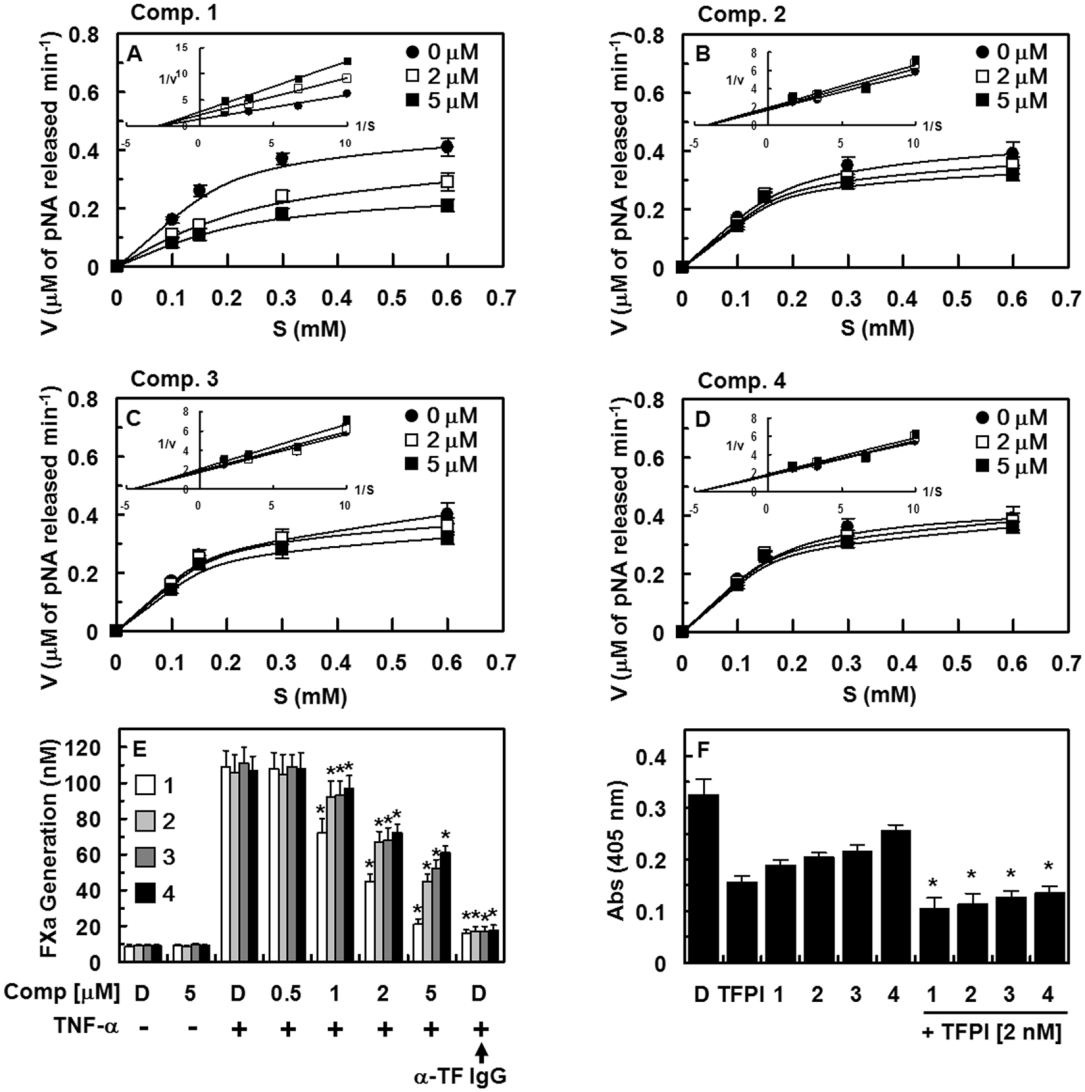
Effects of compounds 1–4 on inhibition and production of FXa. (**A–D**) Michaelis-Menten and Lineweaver-Burk plots (inset) for compounds **1** (**A**), **2** (**B**), **3** (**C**), and **4** (**D**) in PBS at pH 8.34 and 37 °C, demonstrating a noncompetitive inhibition of each compound. The plots represent means of three independent measurements. (**E**) Human umbilical vein endothelial cells (HUVECs) were pre-incubated with the indicated concentration of each compound for 10 min. HUVECs stimulated with tumor necrosis factor-α (TNF-α, 10 ng/mL for 6 h) were incubated with FVIIa (10 nM) and FX (175 nM) in the absence or presence of anti-TF IgG (25 µg/mL). FXa production was determined as described in the “ Supplementary Materials and Methods” section. D = 0.2% DMSO used as the vehicle control. (**F**) Inhibition of FXa by each compound (5 μM) with or without TFPI (2 nM) was monitored using a chromogenic assay. ^*^ *p* < 0.05 vs. TNF-α alone (**E**) or each compound (**F**) alone, analyzed via one-way ANOVA, followed by Tukey multiple comparison testing.

To elucidate the mechanisms responsible for inhibition of coagulation and platelet aggregation by compounds **1**–**4**, inhibition of FXa activity was determined using chromogenic substrates. Compounds **1**–**4** inhibited the catalytic activity of FXa toward its substrate, S-2222, in a dose-dependent manner; the Michaelis-Menten plots are shown in Fig. 6A–D. Inhibition activity plots were obtained for different concentrations of compound **1** and showed a slope of 0.456 for the control without compound **1**, and slopes of 0.707 and 0.957 for compound **1** at concentrations of 2 and 5 µM, respectively. Lineweaver-Burk plots in Fig. 6A (inset) show noncompetitive inhibition of FXa by compound **1**. We observed a decrease in V_max_, with no effect on the K_m_ of FXa toward S-2222 in the presence of compound **1**. The K_i_ value for FXa inhibition toward S-2222 by compound **1** was determined to be 2.96 µM. We also observed a slope of 0.401 for the control without compound **2**, and slopes of 0.442 and 0.465 for compound **2** at concentrations of 2 and 5 µM, respectively. Lineweaver-Burk plots (Fig. 6B (inset)) show a noncompetitive inhibition FXa by compound **2**. We observed a decrease in V_max_, with no effect on the K_m_ of FXa toward S-2222 in the presence of compound **2**. The K_i_ value for S-2222-associated inhibition of FXa by compound **2** was determined to be 4.13 µM. Inhibition activity plots were obtained for different concentrations of compound **3**, showing a slope of 0.388 for the control without compound **3**, and slopes of 0.408 and 0.464 for compound **3** at concentrations of 2 and 5 µM, respectively. Lineweaver-Burk plots (Fig. 6C (inset)) show noncompetitive inhibition of FXa by compound **3**. We observed a decrease in V_max_, with no effect on the K_m_ of FXa toward S-2222 in the presence of compound **3**; the K_i_ was determined to be 4.45 µM. We also observed a slope of 0.363 for the control without compound **4**, and slopes of 0.376 and 0.398 for compound **4** at concentrations of 2 and 5 µM, respectively. Lineweaver-Burk plots (Fig. 6D (inset)) show a noncompetitive inhibition of FXa by compound **4**. We observed a decrease in V_max_, with no effect on the K_m_ of FXa toward S-2222 in the presence of compound **4**. The K_i_ value for S-2222-associated inhibition of FXa by compound **4** was determined to be 4.72 µM.

Production of FXa by FVIIa is dependent on tissue factor (TF) expression in tumor necrosis factor-α (TNF-α)-stimulated HUVECs^22^. Thus, we investigated the effects of compounds **1**–**4** on the production of FXa by FVIIa. HUVECs were stimulated with TNF-α to induce TF expression, causing an 11.9-fold increase in the rate of FX production by FVIIa (109.4 ± 9.2 nM) over that of non-stimulated HUVECs (9.2 ± 0.8 nM). These effects were abrogated by anti-TF IgG (15.9 ± 2.3 nM; Fig. 6E). In addition, pre-incubation with compounds **1**–**4** dose-dependently inhibited FX production by FVIIa, with compound **1** showing the strongest inhibition (Fig. 6E). We next determined whether each compound compete with tissue factor pathway inhibitor (TFPI) in the inhibition of FXa. As shown in Fig. 6F, inhibitory effect of TFPI on FXa activity was further increased in the presence of each compound. Noting that compounds **1**–**4** inhibited the catalytic activity of FXa by a noncompetitive inhibition model (Fig. 6A–D), compounds **1**–**4** may bind to other sites on FXa that are different from the active site of FXa.

### *In Vivo* Effects of Compounds 1–4 in Models of Arterial and Pulmonary Thrombosis

**Figure 7:**
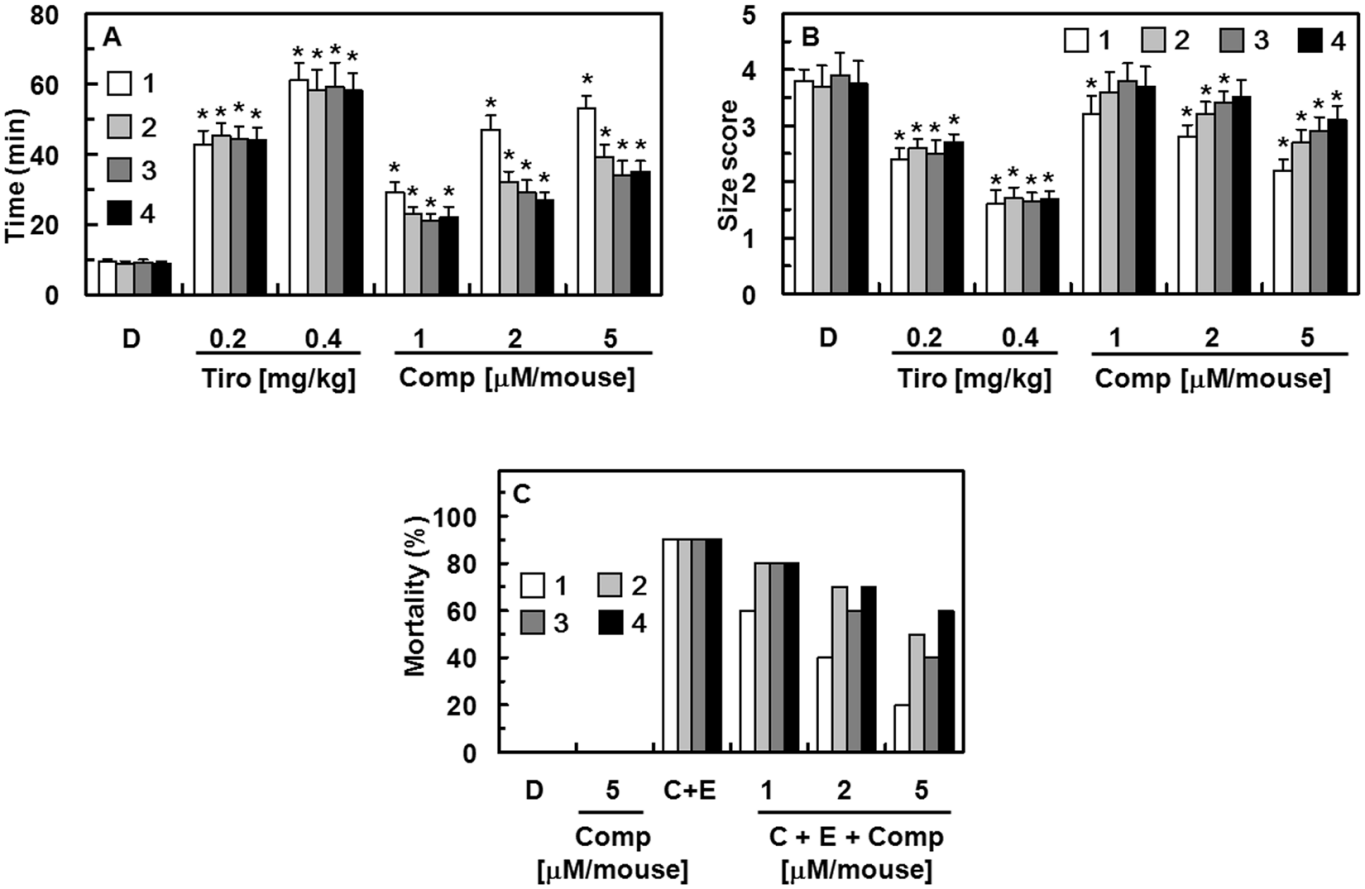
Effects of compounds 1–4 on arterial and pulmonary thrombosis. (**A**) Time to large thrombi formation by compounds **1–4**. Tirofiban (Tiro, 0.2 or 0.4 mg/kg) was used as a positive control. (**B**) Thrombi were scored for size 60 min after FeCl_3_-treatment as described in “Supplementary Materials and Methods.” (**C**) After each compound was injected intravenously, a mixture of collagen (C, 500 µg/kg) and epinephrine (E, 50 µg/kg) was injected into the tail vein of mice to induce acute thrombosis 6 h later. Afterward, mice (20 mice per group) were carefully examined for 15 min to determine whether the mouse was paralyzed, dead, or had recovered from the acute thrombosis challenge. D = 0.2% DMSO used as the vehicle control. *p < 0.05 vs. DMSO, analyzed via one-way ANOVA, followed by Tukey multiple comparison testing.

To examine whether compounds **1**–**4** have antithrombotic and antiplatelet effects *in vivo*, each compound was challenged in a ferric chloride (FeCl_3_)-induced carotid artery thrombosis model ^23^. Tirofiban, a clinical anti-thrombosis drug that acts as a selective glycoprotein IIb/IIIa inhibitor, was used as a positive control. Time to thrombus formation and thrombi size are summarized in Fig. 7. The data show that endothelial injury after FeCl_3_ treatment in control mice leads to growth of large thrombi at 9.3 ± 0.8 min, and tirofiban significantly slowed the growth of large thrombi to 61.1 ± 5.1 min. Compounds **1–4** significantly slowed thrombi growth (Fig. 7A). We also examined the effects of each compound on thrombus size 60 min after FeCl_3_-induced endothelial injury (Fig. 7B). The results show that compounds **1**–**4** reduce FeCl_3_-induced thrombus formation. The results from the *in vivo* pulmonary thrombosis model are shown in Fig. 7C. A mixture of collagen and epinephrine that was injected intravenously into mice induced massive pulmonary thromboses, causing acute paralysis and sudden death (90% mortality). Mortality was significantly lower in mice co-treated with compounds **1**–**4** than that of mice treated with the mixture of collagen and epinephrine only (Fig. 7C).

### *In Vivo* Effects of Compounds 1–4 on Bleeding Time

To assess bleeding risk incurred by compounds **1**–**4**, we measured bleeding time in mice treated with each compound (up to 30 µM/mouse) by using a tail-cutting assay; the concentration used was three-fold higher than the doses used for the *in vivo* anti-thrombotic studies. Aspirin-treated mice (9 mg/kg) served as positive controls. As shown in Fig. 4C, a slight prolongation of bleeding time was observed in mice treated with 30 µM (14.3 ± 1.2, 13.5 ± 1.1, and 12.3 ± 1.3 min for compounds **1**, **2**, and **3**, respectively, mean ± SD, n = 10). However, the bleeding time in mice treated with compounds **1**–**4** was shorter than that observed in mice treated with aspirin (18.2 ± 1.6 min, mean ± SD, n = 10). The dose required to avoid thrombus formation and to protect against paralysis or death in mice was determined to be 5 µM per mouse (Fig. 7C). At double this concentration (i.e., 10 µM), bleeding time was not significantly longer in mice treated with compounds **1**–**4** than that of mice treated with vehicle (13.5 ± 1.3, 12.5 ± 1.2, and 12.1 ± 1.1 min for compounds **1**, **2**, and **3**, respectively, mean ± SD, n = 10), suggesting that compounds **1**–**4** confer a low bleeding risk.

## Discussion

Although several anticoagulants such as fondaparinux, warfarin, low-molecular-weight heparins (LMWHs), and unfractionated heparin are effective in the prevention and treatment of thrombotic diseases, these drugs also have undesirable effects^24, 25^. In the past decades, numerous research efforts were made to identify novel anticoagulants with improved efficacy and safety. After extensive investigation, FXa became a promising target for the development of potent and selective anticoagulants, especially since FXa is situated at the beginning of the intrinsic and extrinsic coagulation pathways^6, 7^.

In this study, compounds **1–5** were isolated from *O. chinensis sinuosa*, and their antithrombotic and antiplatelet activity was determined. Compounds **1** and **2** were isolated for the first time from a natural source, while compounds **3**–**5** have been reported previously^18, 20^. The absolute configuration of the 1,4-dioxane ring in the *N*-acetyldopamine dimers was found to be (2 *R*, 3 *S*) or (2 *S*, 3 *R*). In contrast to the reported *N*-acetyldopamine dimers, compounds **1** and **2** are specific diastereomers with 2 *R* and 3 *R* absolute configuration. *N*-acetyldopamine dimers have been reported to have diverse bioactivities, such as 2,2-diphenyl-1-picrylhydrazyl (DPPH) radical scavenging activity and anti-inflammatory effects related to nuclear factor-κB (NF-κB), inducible nitric oxide synthase (iNOS), interleukin (IL)-6, TNF-α, and cyclooxygenase (COX)-2 in LPS-stimulated RAW264.7 cells^20^. In addition, tyrosinase and COX-2 inhibitory activities of *N*-acetyldopamine dimers have been reported^26^. However, this is the first report of the antithrombotic activity of *N*-acetyldopamine dimers. Compounds **1** and **2**, with 2 *R*, 3 *R* absolute configuration on the 1,4-dioxane ring of the *N*-acetyldopamine dimer, displayed stronger antithrombotic activity than compounds **3** and **4** with 2 *R*, 3 *S* absolute structure. Compound **5**, which bears one double bond in the *N*-acetylamino-2-ethyl moiety, did not show antithrombotic activity. This result suggests that flexibility of the *N*-acetylamino-2-ethyl group on the dibenzo-1,4-dioxane structure is essential to the activity. Compounds **1**–**4** showed FXa inhibitory and anti-platelet aggregation activities and showed potent antithrombotic activity using *in vivo* models of arterial and pulmonary thrombosis. Interestingly, these compounds did not prolong bleeding times in mice at effective antithrombotic doses, indicating a favorable efficacy to bleeding ratio.

FXa is a trypsin-like serine protease that plays a pivotal role in the blood coagulation cascade^2^. During activation of the coagulation cascade, FXa forms a complex with calcium ions and FVa on the platelet membrane and converts prothrombin to thrombin^2^. Noting that direct thrombin inhibitors have a narrow therapeutic index, we hypothesized that selective inhibition of FXa is a potential and promising strategy for the design of new antithrombotic agents, especially since FXa is positioned at the beginning of the common extrinsic and intrinsic coagulation pathways^6, 7^. Many FXa inhibitors isolated from animals have molecular weights in the 1–29 kDa range^27–30^, but the molecular weights of compounds **1**–**4** (molecular weight of 386.15) are smaller than any other known FXa inhibitors. Therefore, compounds **1**–**4** would have advantages, such as low immunogenicity and low production costs, compared to relatively large antithrombotic proteins or peptides. Platelet aggregation assays and clotting time measurements are the most commonly used methods to determine the efficacy of new anti-thrombotic drugs^31^. The anticoagulant effects of compounds **1–4** were verified with both an aPTT and PT *ex vivo* assay. Compounds **1–4** are also selective platelet aggregation inhibitors, as shown by inhibition of ADP- and U46619-induced, but not thrombin-induced, platelet aggregation. These data confirm that compounds **1–4** do not inhibit the activity or production of thrombin under these conditions (data not shown).

Compounds **1–4** showed similar anti-thrombotic efficacy to rivaroxaban (a direct FXa inhibitor) such as increased blood clotting time, delayed thrombogenesis and thrombogenic time, and inhibition of the production and activity of FXa. Although rivaroxaban did not affect platelet aggregation induced by ADP, thrombin, or U46619^32^, compounds **1–4** effectively and concentration-dependently inhibited ADP- or U46619-induced platelet aggregation. Further, rivaroxaban prolonged the generation of thrombin and reduced the thrombin burst produced in the propagation phase^32^ whereas compounds **1–4** did not affect the generation and activity of thrombin. Furthermore, the bleeding times were not significantly affected by rivaroxaban at antithrombotic doses below the ED_50_ required for antithrombotic efficacy in the bleeding time models^32, 33^. At higher doses of rivaroxaban in the rat tail-bleeding time model, bleeding times were dose-dependently prolonged^32, 33^. However, the mouse tail-bleeding time was not affected by compounds **1–4**. Therefore, as each compound selectively inhibited FXa amidolytic activity without affecting the function of thrombin in the circulation system, it should have a minimal effect on normal hemostatic responses and regulatory processes, indicating that compounds **1–4** have favorable benefits compared to rivaroxaban. Furthermore, a noncompetitive inhibitory mechanism of compounds **1–4** on FXa activity was proved by enhanced inhibitory effects of TFPI on FXa activity in the presence of each compound.

A characteristic of the FeCl_3_-induced thrombosis mouse model is the formation of thrombi and large platelet aggregates that are surrounded by erythrocytes and fibrin^23, 34^. FXa binds to clots during clot formation and contributes to the procoagulant growth of thrombi^35, 36^. When associated with thrombi, FXa is resistant to inhibition by thrombin-dependent anticoagulants^35^. In this study, compounds **1**–**4** suppressed the formation of stable occlusive thrombi that quickly form in the FeCl_3_-injury model (Fig. 7). Therefore, the inhibition of clot-associated FXa by direct (i.e., thrombin-independent) FXa inhibitors seems more effective than by indirect (i.e., thrombin-dependent) FXa inhibitors for the prevention of thrombosis.

In conclusion, we suggest that the *N*-acetyldopamine dimers **1–4**, in particular two new diastereomers **1** and **2**, from the edible insect *O. chinensis sinuosa* are novel antithrombotic compounds capable of inhibiting intrinsic blood coagulation pathways via FXa. They also inhibit agonist-induced platelet aggregation, showing potent antithrombotic activity *in vivo* without an obvious bleeding risk. These results may be of interest to those designing pharmacological strategies for the treatment or prevention of coagulation-related vascular diseases.

## Methods

### Reagents

Freeze-dried *Oxya chinensis sinuosa* (5 kg) were obtained from the National Academy of Agricultural Science, RDA, Korea, and identified by one of the authors (M-A. Kim). A voucher specimen (CNU-INS 201603) was deposited at the Pharmacognosy Laboratory of the College of Pharmacy, Chungnam National University, Daejeon, Korea. TNF-α was purchased from Abnova (Taipei, Taiwan). The anti-tissue factor (TF) antibody was purchased from Santa Cruz Biotechnology, Inc. (Santa Cruz, CA, USA). The thromboxane A2 (TXA2) analog, U46619, was purchased from Calbiochem-Novabiochem Corp. (San Diego, CA, USA). Factors VIIa, X, and Xa, and thrombin were obtained from Haematologic Technologies (Essex Junction, VT, USA). Recombinant tissue factor pathway inhibitor (TFPI) was obtained from Amercian Diagnostica Inc (Stamford, CT, USA). The aPTT assay reagent and PT reagents were purchased from Fisher Diagnostics (Middletown, VA, USA); the chromogenic substrate (S-2222) was purchased from Chromogenix AB (Mölndal, Sweden). Collagen and tirofiban were purchased from Sigma (St. Louis, MO, USA). All other reagents were of the highest commercially available grade.

### General experimental procedures

Nuclear magnetic resonance (NMR) experiments were carried out using a Bruker DMX 600 (^1^H-600 MHz, ^13^C-150 MHz) spectrometers. Thin layer chromatography (TLC) was run on glass plates precoated with silica gel 60 F_254_ and RP-18 F_254_ (20 × 20 cm, 200 *μ*m, 60 Å, Merck). Vacuum liquid chromatography (VLC) was conducted on Merck silica gel (70–230 mesh), and medium pressure liquid chromatography (MPLC) was performed utilizing a Biotage Isolera apparatus equipped with a reversed-phase C_18_ SNAP Cartridge KPC18-HS (340 g, Biotage AB, Uppsala, Sweden). Preparative reversed phase HPLC (prep RP-HPLC) separation was performed on a Gilson system with UV detector, using a Phenomenex Kinetex C_18_ column (250 × 21.2 mm, 5 *μ*m, Phenomenex, Torrance, CA, USA), Kinetex Biphenyl column (250 × 21.2 mm, 5 *μ*m), or YMC column (250 × 20.0 mm, 5 *μ*m) at 5 mL/min flow rate. Optical rotations were measured on a JASCO DIP-370 (Tokyo, Japan) automatic digital polarimeter. Circular dichroism (CD) data were measured at 23 °C with a J815 spectrometer, using 1-mm light path cuvettes (Jasco) and concentrations of 0.5 mg/mL in various pH buffers. Wavelength scans were monitored from 300 to 200 nm with an average of five measurements. High-resolution electrospray ionization mass spectrometry (HRESIMS) data were obtained on a AB SCIEX TripleTOF™ 5600 high-resolution mass spectrometer (SCIEX). Electrospray ionization mass spectrometry (ESIMS) data were obtained on a SYNAPT G2 Waters mass spectrometer (Manchester, UK). HPLC-evaporative light scattering detector (ELSD) data were acquired on a Shimadzu LC-10AD series system (Kyoto, Japan), with a Sedex 55 ELSD detector at 25 °C.

### Extraction and isolation

The freeze-dried whole bodies of *Oxya chinensis sinuosa* (5 kg, powder) were refluxed three times with 1% acetic acid in ethanol (5 L × 4). The extract was concentrated under vacuum to yield a brownish ethanol extract (421.8 g). The ethanol extract (419 g) was separated by VLC (20 cm × 20 cm), eluting with a stepwise gradient of *n*-hexane:ethyl acetate (100:0, 80:20, 60:40, 40:60, and 20:80), and a stepwise gradient of chloroform:methanol (100:0, 80:20, 60:40, 40:60, 20:80, and 0:100) to yield 12 fractions. According to the TLC profiles, the 12 fractions were consolidated to 6 fractions (A-F). Fraction D (35.7 g) that eluted with *n*-hexane:ethyl acetate (from 60:40 to 40:60) was further subjected to VLC, eluting with a stepwise gradient of *n*-hexane:ethyl acetate (100:0, 85:15, 75:25, and 50:50) and chloroform:methanol (85:15, 80:20, 67:33, 50:50, and 0:100) to obtain 6 subfractions (Fr. D1-6). Fraction D-5 (18.3 g) that eluted with chloroform:methanol (from 80:20 to 67:33) was subjected to MPLC [column: C_18_ SNAP Cartridge KPC18-HS (340 g); mobile phase: acetonitrile:H_2_O (linear gradient from 10:90 to 60:40, 8160 mL)] to yield 6 fractions (D5-1-6). Fraction D-5-2 (1.9 g) was subjected to preparative HPLC (Phenomenex Kinetex C_18_ column (250 × 21.2 mm, 5 *μ*m), using a gradient of acetonitrile in water (from 20% acetonitrile, 0 min to 25%, for 70 min) to afford compounds **3** (*t*_*R*_ 52 min, 331.2 mg), **4** (*t*_*R*_ 47 min, 389.2 mg), and **5** (*t*_*R*_ 64 min, 37.2 mg). Novel compounds **1** (*t*_*R*_ 43 min, 8.9 mg) and **2** (*t*_*R*_ 42 min, 13.6 mg) were further purified from subfraction D-5-2-57 min (35.8 mg) by preparative HPLC on a Kinetex Biphenyl column (250 × 21.2 mm, 5 *μ*m), using a gradient of methanol in water from 30% to 80% over 60 min.

### Animals and husbandry

Male C57BL/6 mice (6–7 weeks, 27 g) were purchased from Orient Bio Co. (Sungnam, Republic of Korea) and were used after a 12-day acclimatization period. The mice were housed at five per polycarbonate cage under a controlled temperature (20–25 °C) and humidity (40–45% relative humidity) with a 12:12 h light:dark cycle. They received normal rodent pellet diet and water *ad libitum* during acclimatization. They were treated in accordance with the Guidelines for the Care and Use of Laboratory Animals issued by Kyungpook National University, Republic of Korea (IRB No. KNU 2016-54).

### *Ex vivo* coagulation assay

Fifteen min after the administration of each compound or saline, blood was sampled, and plasma was obtained by centrifuging at 2,000 × *g* for 10 min at room temperature in order to measure prothrombin time (PT) and activated partial thromboplastin time (aPTT). The aPTT and PT were determined using a Thrombotimer (Behnk Elektronik, Norderstedt, Germany), according to the manufacturer’s instructions and as described previously^37^. Briefly, platelet-poor plasma (PPP, 100 μL) was mixed with aPTT assay reagent (100 μL) for 1 min at 37 °C, followed by the addition of 20 mM CaCl_2_ (100 μL). The clotting times were recorded. For the PT assays, PT assay reagent (200 μL) was incubated for 10 min at 37 °C. And, PPP (100 μL) was mixed with PT assay reagent (200 μL) for 3 min at 37 °C and the clotting time was recorded. All experimental protocols (KNUH 2012-01-010) were approved by the Institutional Review Board of Kyungpook National University Hospitals (Daegu, Republic of Korea).

### Bleeding time

Tail bleeding times were measured using the method described by Dejana *et al*.^37^. Briefly, C57BL/6 mice were fasted overnight prior to the experiments. One hour after the i.v. administration of each compound, the tails of the mice were transected 2 mm from their tips. Bleeding time was defined as the time elapsed until bleeding stopped. Bleeding times that exceeding 15 min were recorded as 15 min.

### *In vitro* platelet aggregation assay

The *in vitro* platelet aggregation assay was performed according to a previously reported method^38, 39^. Platelet rich plasma (PRP) was incubated with the indicated concentration of each compound in DMSO for 1, 3, 5, or 10 min. Samples were subsequently stimulated by ADP (10 μM), U46619 (6 μM), or thrombin (3 U/mL). Platelet aggregation was recorded using an aggregometer (Chronolog, Havertown, PA, USA).

### Inhibition of amidolytic activity of FXa

Each compound with or without TFPI was dissolved in 50 mM Tris-HCl buffer (pH 7.4) containing 7.5 mM EDTA and 150 mM NaCl. Following a 2-min incubation at 37 °C, FXa solution (150 μL, 1 U/mL) was added, followed by incubation at 37 °C for 1 min. S-2222 (an FXa substrate, 150 μL, 1.5 mM) solution was subsequently added, and the absorbance at 405 nm was monitored for 20 min using a spectrophotometer (TECAN, Männedorf, Switzerland). The absorbance-time curve and the slope of the curve (V_i_) were used to represent the activity of the enzyme. Distilled water served as a control (the slope of curve V_0_). The inhibitory effect was calculated according to equation (1):

1 $${\rm{Inhibitory}}\,{\rm{rate}}( \% )=({{\rm{V}}}_{{\rm{0}}}-{{\rm{V}}}_{{\rm{i}}})/{{\rm{V}}}_{0}\times 100$$

where V_0_ represents the slope of the vehicle and V_i_ represents the slope of the samples.

### Determination of the inhibitory constant for FXa and enzyme inhibition

The inhibitor (I) constants (K_i_) were determined for the inhibition of a series of human enzymes (FXa, thrombin, trypsin, plasmin, protein Ca, streptokinase, tPA, and urokinase) by each compound. Chromogenic substrate assays were performed using a Labsystems IEMS (Cergy Pontoise, France) microtiter plate reader. In each assay, the compound was tested at a minimum of seven concentrations in duplicate to obtain an inhibition curve. Assays were performed according to the following general procedure. In a 96-well microtiter plate, 25 μL of compound, inhibitor solution or buffer was added to 50 μL of substrate. A volume of 25 μL of enzyme solution was added just before the plate was placed in the microtiter plate reader for 1 h at 37 °C. The hydrolysis of the substrate yields *p*-nitroaniline, which was continuously monitored spectrophotometrically at 405 nm. Data were collected and the initial rate of substrate hydrolysis [V_o_ (mOD/minute)] was calculated. Following Michaelis-Menten kinetics, the affinity of the enzyme for the substrate, in the absence of the inhibitor (K_m_) and in the presence of an inhibitor (K_p_), was determined to be the negative inverse x-intercept of Lineweaver–Burk plots. The dissociation constant for inhibition (K_i_) was calculated using the following equation: K_i_ = (K_m_·[I])/(K_p_ − K_m_) as described by Williams and Morrison^40^.

### Cell culture

Primary HUVECs were obtained from Cambrex Bio Science (Charles City, IA, USA) and were maintained as described previously^41, 42^. Briefly, cells were cultured in EBM-2 basal media supplemented with growth supplements (Cambrex Bio Science, Charles City, IA, USA) at 37 °C under a 5% CO_2_ atmosphere until confluent. All experiments were performed with HUVECs at passage 3–5.

### Production of factor Xa on the surface of HUVECs

The TNF-α-stimulated (10 ng/mL for 6 h in serum-free medium), confluent monolayer of HUVECs (preincubated with the indicated concentrations of each compound for 10 min) in a 96-well culture plate was incubated with FVIIa (10 nM) in buffer B (buffer A [10 mM HEPES, pH 7.45, 150 mM NaCl, 4 mM KCl, and 11 mM glucose] with 5 mg/mL bovine serum albumin [BSA] and 5 mM CaCl_2_) for 5 min at 37 °C in the presence or absence of anti-TF IgG (25 μg/mL). FX (175 nM) was subsequently added to the cells in a final reaction volume of 100 μL, and the cells were incubated for 15 min. The reaction was stopped by the addition of buffer A containing 10 mM EDTA, and the FXa generated was measured using a chromogenic substrate. The changes in absorbance at 405 nm over 2 min were monitored using a microplate reader (Tecan Austria GmbH, Grödig, Austria). The initial color development rates were converted into FXa concentrations using a standard curve prepared with known dilutions of purified human FXa.

### Arterial thrombosis animal model

The FeCl_3_-induced thrombosis mouse model was established as previously described^23^. Male C57BL/6 mice were fasted overnight and were administered each indicated compound in DMSO by intravenous injection. Mice were then anesthetized with 3% isoflurane (Forane®, Choongwae Pharma. Corp., Seoul, Korea) and injected intravenously with 0.1 mL of 0.1% rhodamine 6 G (Sigma). A testicular artery (200 μm in diameter) was carefully exposed and a cotton thread (0.2 mm in diameter) saturated with 0.25 M FeCl_3_ was applied to the adventitial surface. After 5 min, the cotton thread was removed, and the wound was flushed with saline solution. Thrombus formation was monitored at 35 °C by 3D imaging as previously described^43^. The size and time of thrombus formation were monitored, and the findings were categorized as follows: score 0 indicated no thrombus; 1 indicated a small thrombus (50 μm × 75 μm); 2 indicated a medium-sized thrombus (100 μm × 150 μm); and 3 indicated a large thrombus (200 μm × 300 μm). The time from FeCl_3_-mediated endothelial injury to occlusion of the testicular artery by a large thrombus was also recorded.

### Acute thrombosis induced by a combination of collagen and epinephrine in mice

Male C57BL/6 mice were fasted overnight and divided into groups of 10 animals. Each compound, suspended in DMSO, was administered to mice intravenously. A mixture of collagen (500 μg/kg) plus epinephrine (50 μg/kg) was injected into the tail vein of mice to induce acute thrombosis 6 h later. Each mouse was carefully examined for 15 min to determine whether the mouse was paralyzed, dead, or had recovered from the acute thrombotic challenge. For statistical analysis, five separate experiments were performed.

### Statistical analysis

The results were expressed as means ± standard deviations (SD) of at least three independent experiments performed in duplicate. *P* < 0.05 was considered statistically significant and was determined using SPSS software (version 14.0, SPSS Science, Chicago, IL, USA). Statistical differences were determined by one-way analysis of variance (ANOVA) and Tukey’s post-test.

## Electronic supplementary material

Supplementary information
