# Supplementary material for: Evaluation of novel factor Xa inhibitors from Oxya chinensis sinuosa with anti-platelet aggregation activity
Source: Sci Rep. 2017 Aug 11;7:7934. doi: 10.1038/s41598-017-08330-1 (PMC5554137; doi:10.1038/s41598-017-08330-1)
Supplement: Supplementary file 1 — Supplementary information [file 41598_2017_8330_MOESM1_ESM.doc]

**Evaluation of novel factor Xa inhibitors from *Oxya chinensis sinuosa* with anti-platelet aggregation activity**

Wonhwa Lee ^1†^, HeeSeung Lee^2†^, Mi-Ae Kim^3†^, Joonhyeok Choi^1^, Kyung-Min Kim^4^, Jae Sam Hwang^3^, MinKyun Na^2^*, and Jong-Sup Bae^1^*

^1^College of Pharmacy, CMRI, Research Institute of Pharmaceutical Sciences, BK21 Plus KNU Multi-Omics based Creative Drug Research Team, Kyungpook National University, Daegu 41566, Republic of Korea; ^2^College of Pharmacy, Chungnam National University, Daejeon 34134, Republic of Korea; ^3^Department of Agricultural Biology, The National Academy of Agricultural Science, RDA, Wanju-gun 55365, Republic of Korea; ^4^Division of Plant Biosciences, School of Applied BioSciences, College of Agriculture and Life Science, Kyungpook National University, Daegu 41566 Republic of Korea

Running title: Antithrombotic effects of *N*-acetyldopamine dimers

^†^These authors contributed equally to this work

* Corresponding Authors:

MinKyun Na, Ph.D.

College of Pharmacy, Chungnam National University

99 Daehak-ro, Yuseong, Daejeon 34134, Republic of Korea

Phone, 82-42-821-5925; Fax, 82-42-823-6566; Email, mkna@cnu.ac.kr

and

Jong-Sup Bae, Ph.D.

College of Pharmacy, Kyungpook National University

80 Daehak-ro, Buk-gu, Daegu 41566, Republic of Korea

Phone, 82-53-950-8570; Fax, 82-53-950-8557; Email, baejs@knu.ac.kr

Contents

[**Figure S1.** HRESIMS spectrum of compound 1](#__RefHeading___Toc462241733)

[**Figure S2.** ^1^H NMR of compound 1 (600 MHz, methanol-*d_4_*)](#__RefHeading___Toc462241734)

[**Figure S3.** ^13^C NMR spectrum of compound **1** (150 MHz, methanol-*d_4_*)](#__RefHeading___Toc462241737)

[**Figure S4.** HSQC data of compound 1](#__RefHeading___Toc462241738)

[**Figure S5.** HMBC data of compound 1](#__RefHeading___Toc462241739)

[**Figure S6.** The CD spectrum of compound 1](#__RefHeading___Toc462241740)

[**Figure S7.** HRESIMS spectrum of compound 2](#__RefHeading___Toc462241741)

[**Figure S8.** ^1^H NMR of compound 2 (600 MHz, methanol-*d_4_*)](#__RefHeading___Toc462241742)

[**Figure S9.** ^13^C NMR spectrum of compound **2** (150 MHz, methanol-*d_4_*)](#__RefHeading___Toc462241743)

[**Figure S10.** HSQC data of compound 2](#__RefHeading___Toc462241744)

[**Figure S11.** HMBC data of compound 2](#__RefHeading___Toc462241745)

[**Figure S12.** The CD spectrum of compound 2](#__RefHeading___Toc462241746)

[**Figure S13.** ESIMS spectrum of compound 3](#__RefHeading___Toc462241747)

[**Figure S14.** ^1^H NMR of compound 3 (600 MHz, methanol-*d_4_*)](#__RefHeading___Toc462241751)

[**Figure S15.** ^13^C NMR spectrum of compound **3** (150 MHz, methanol-*d_4_*)](#__RefHeading___Toc462241754)

[**Figure S16.** The CD spectrum of compound 3](#__RefHeading___Toc462241757)

[**Figure S17.** ESIMS spectrum of compound 4](#__RefHeading___Toc462241758)

[**Figure S18.** ^1^H NMR of compound 4 (600 MHz, methanol-*d_4_*)](#__RefHeading___Toc462241759)

[**Figure S19.** ^13^C NMR spectrum of compound **4** (150 MHz, methanol-*d_4_*)](#__RefHeading___Toc462241760)

[**Figure S20.** The CD spectrum of compound 4](#__RefHeading___Toc462241761)

[**Figure S21.** ESIMS spectrum of compound 5](#__RefHeading___Toc462241762)

[**Figure S22.** ^1^H NMR of compound 5 (600 MHz, methanol-*d_4_*)](#__RefHeading___Toc462241763)

[**Figure S23.** ^13^C NMR spectrum of compound **5** (150 MHz, methanol-*d_4_*)](#__RefHeading___Toc462241764)

[**Figure S24.** The CD spectrum of compound 5](#__RefHeading___Toc462241765)

[**Figure S25.** HPLC-ELSD profiling of compounds 1-5](#__RefHeading___Toc462241766)

Found elemental compositions

|  | **Hit** | **Formula** | **Theoretical m/z** | **ppm** |
| --- | --- | --- | --- | --- |
| **409.1346** | 1 | NaC_20_H_22_N_2_O_6_ | 409.1376 | -7.3 |

**Figure S1.** HRESIMS spectrum of compound **1**


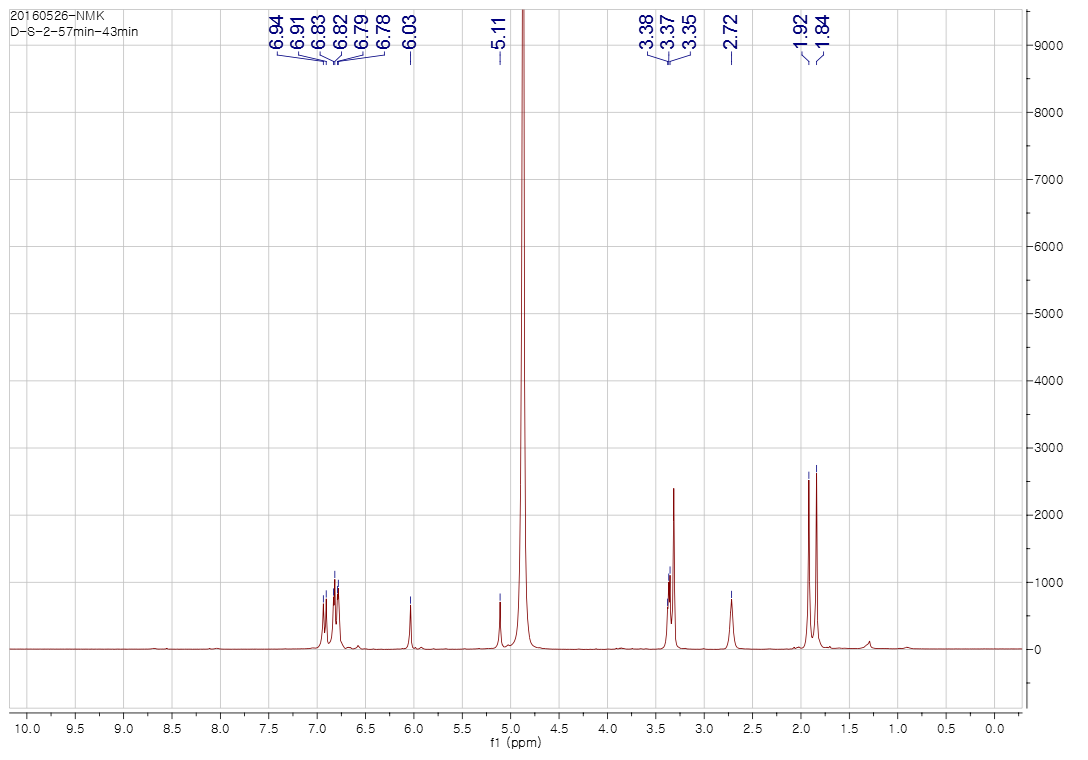


**Figure S2.** ^1^H NMR of compound **1** (600 MHz, methanol-*d_4_*)


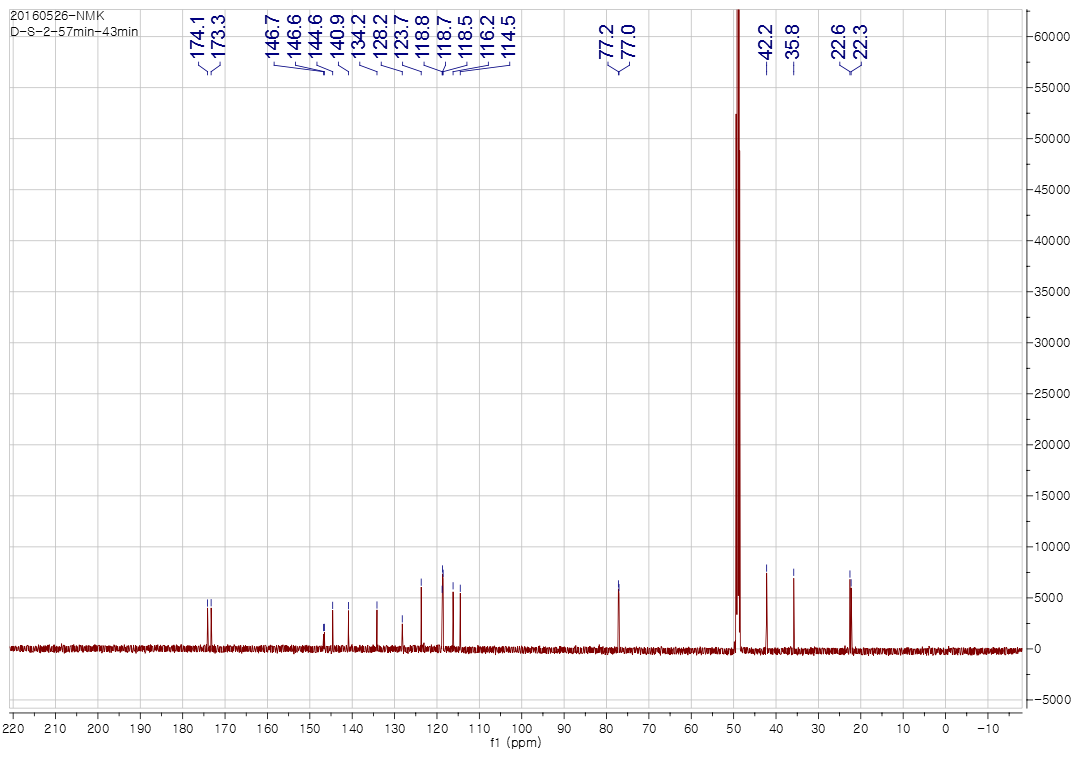


**Figure S3**. ^13^C NMR spectrum of compound **1** (150 MHz, methanol-*d_4_*)


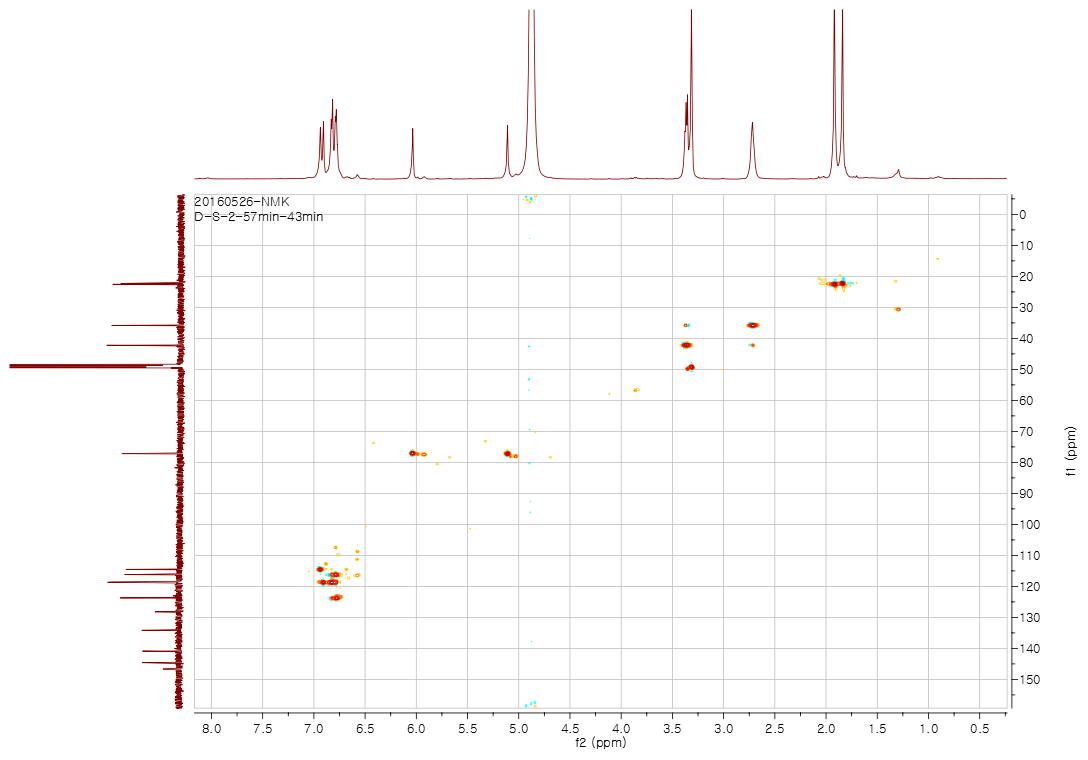


**Figure S4.** HSQC data of compound **1**


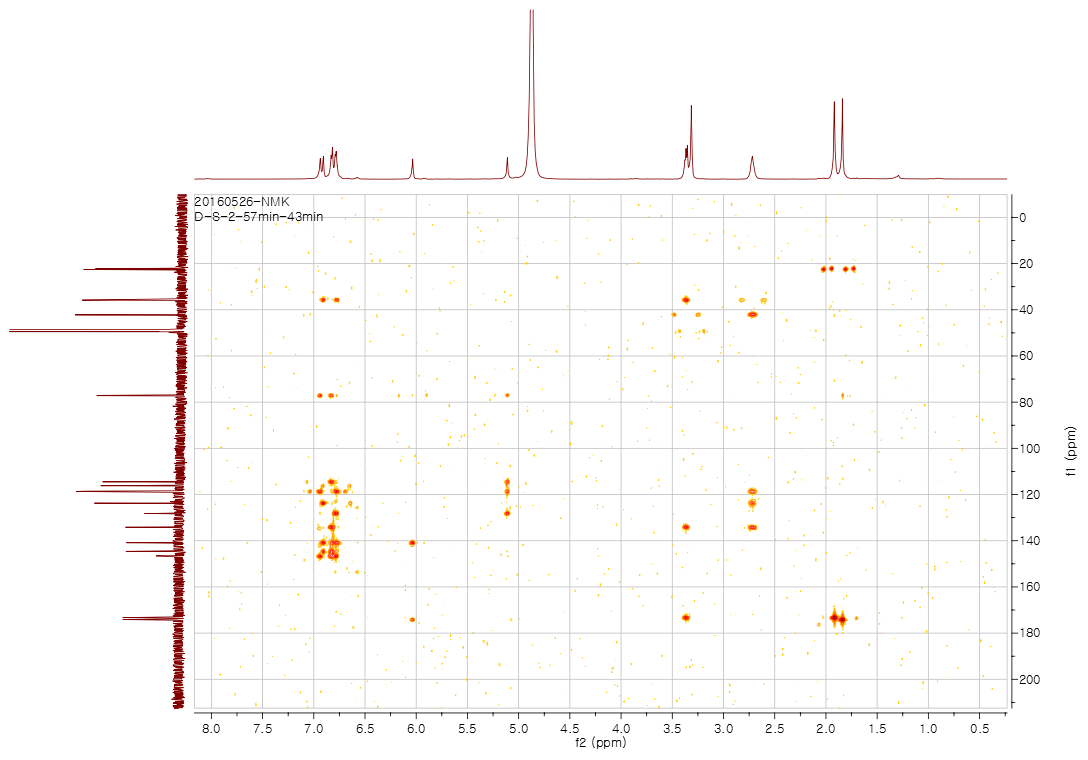


**Figure S5.** HMBC data of compound **1**


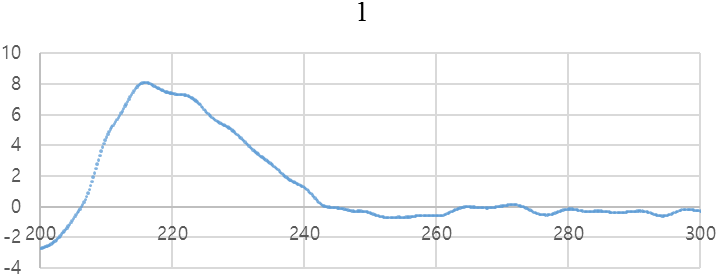


**Figure S6.** The CD spectrum of compound **1**

Found elemental compositions

|  | **Hit** | **Formula** | **Theoretical m/z** |  | | **ppm** | | **MS Rank** |  | |
| --- | --- | --- | --- | --- | --- | --- | --- | --- | --- | --- |
| **409.1345** | 1 | NaC_20_H_22_N_2_O_6_ | 409.1376 | |  | | -7.6 | 1 |  |  |

**Figure S7.** HRESIMS spectrum of compound **2**


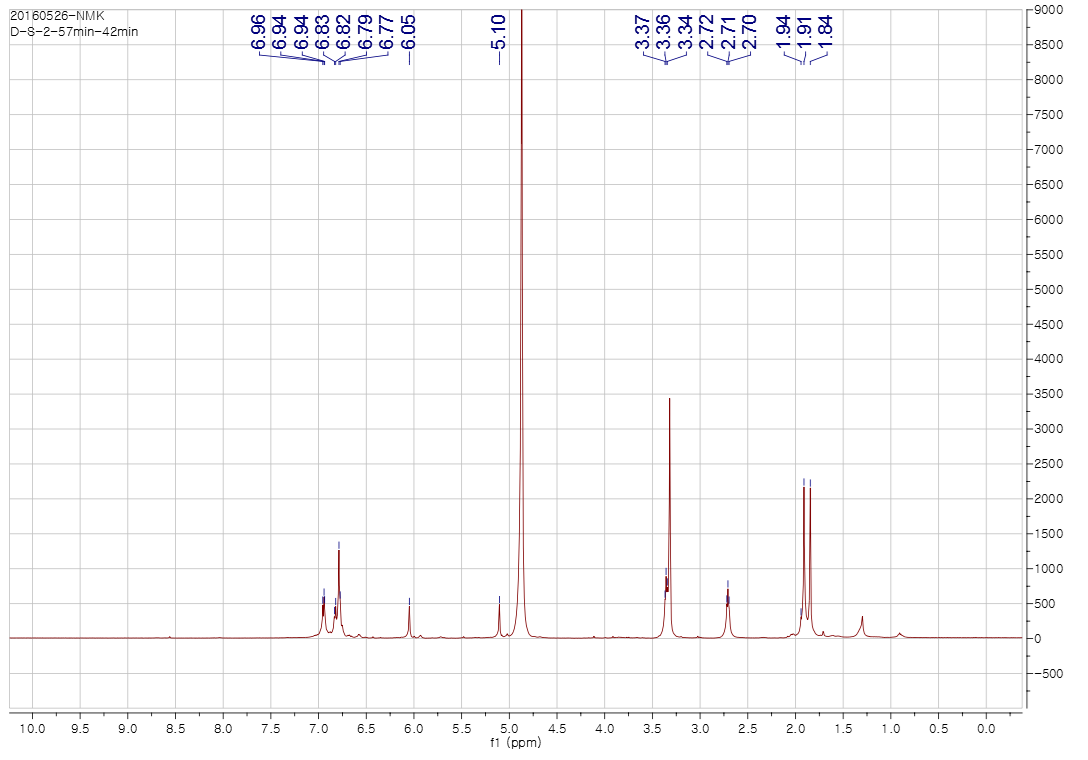


**Figure S8.** ^1^H NMR of compound **2** (600 MHz, methanol-*d_4_*)


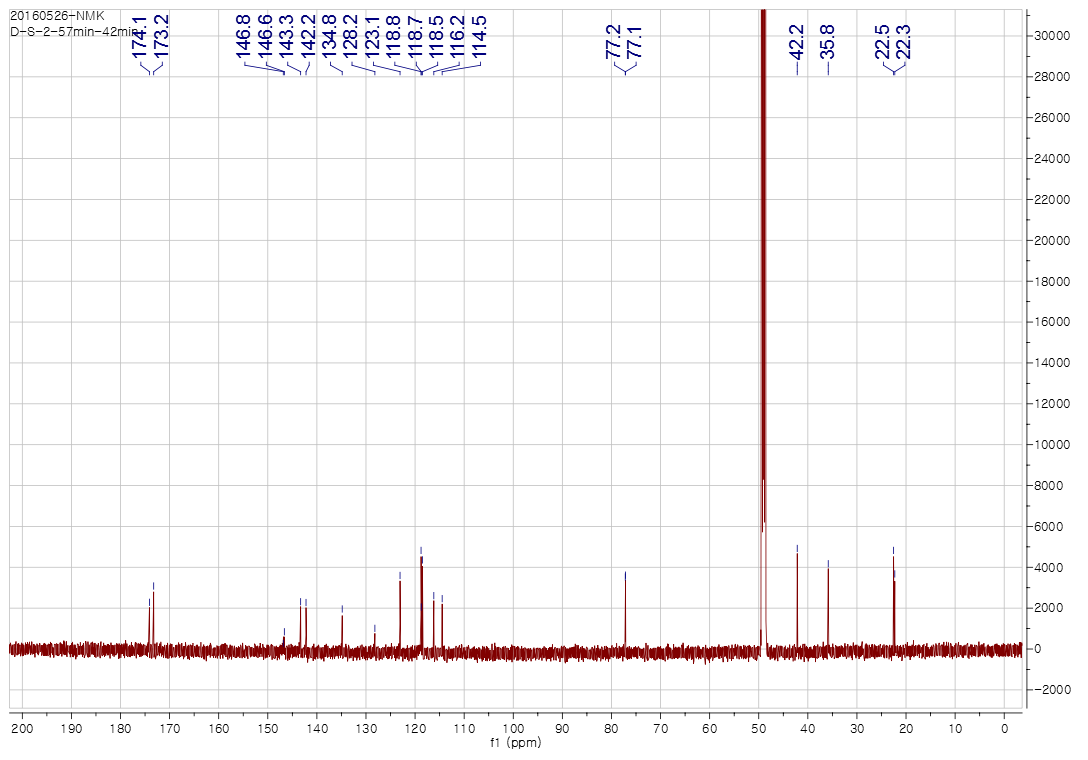


**Figure S9**. ^13^C NMR spectrum of compound **2** (150 MHz, methanol-*d_4_*)


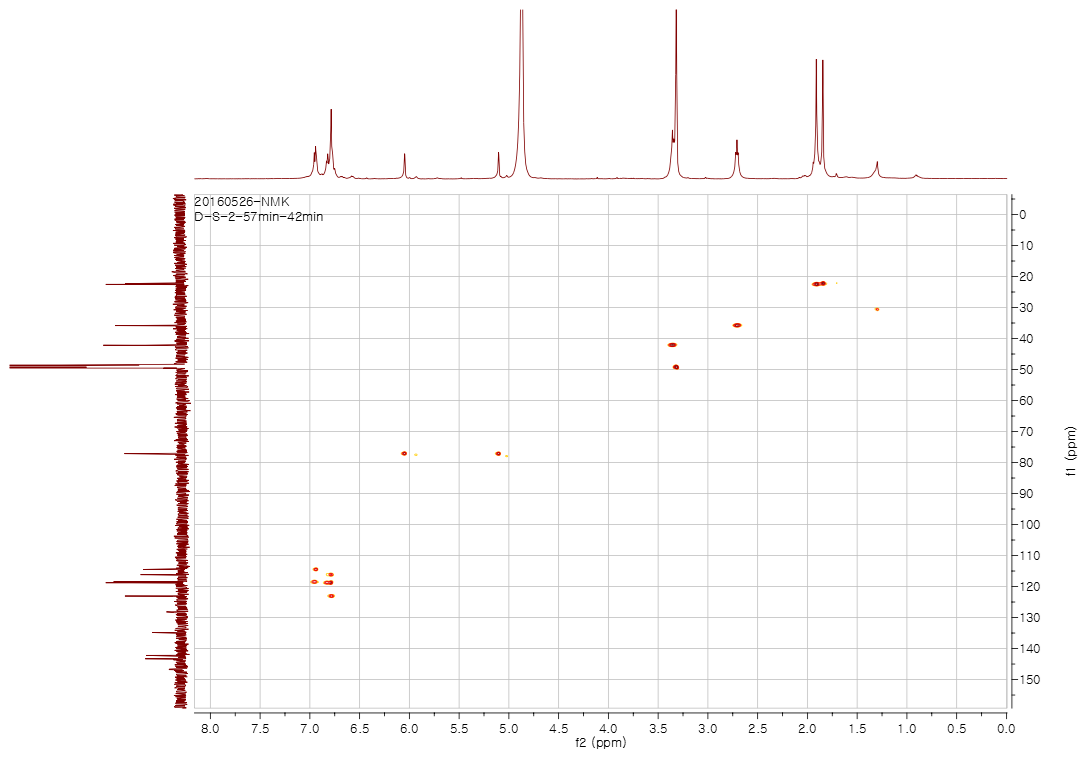


**Figure S10.** HSQC data of compound **2**


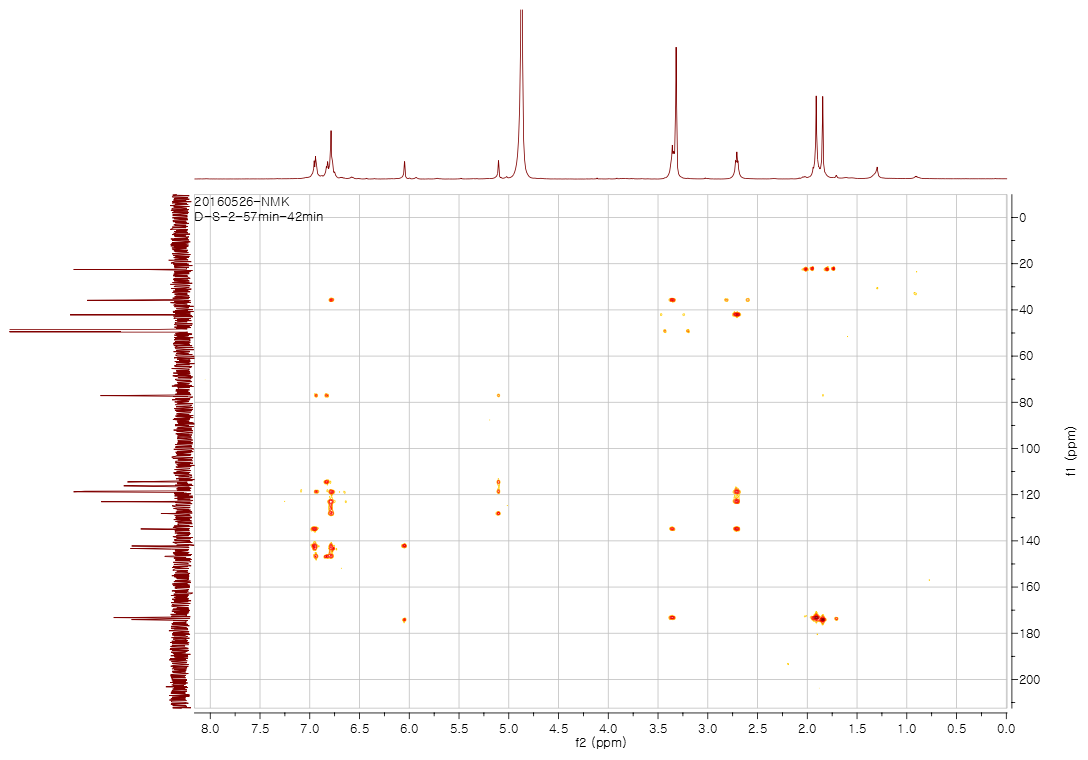


**Figure S11.** HMBC data of compound **2**


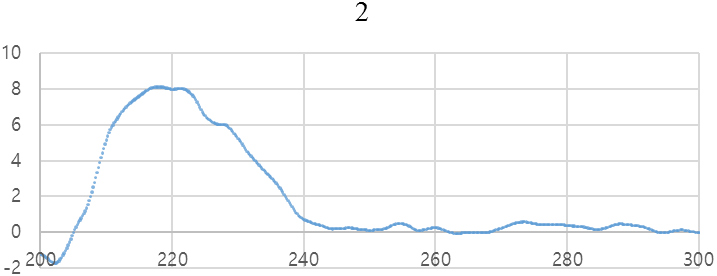


**Figure S12.** The CD spectrum of compound **2**


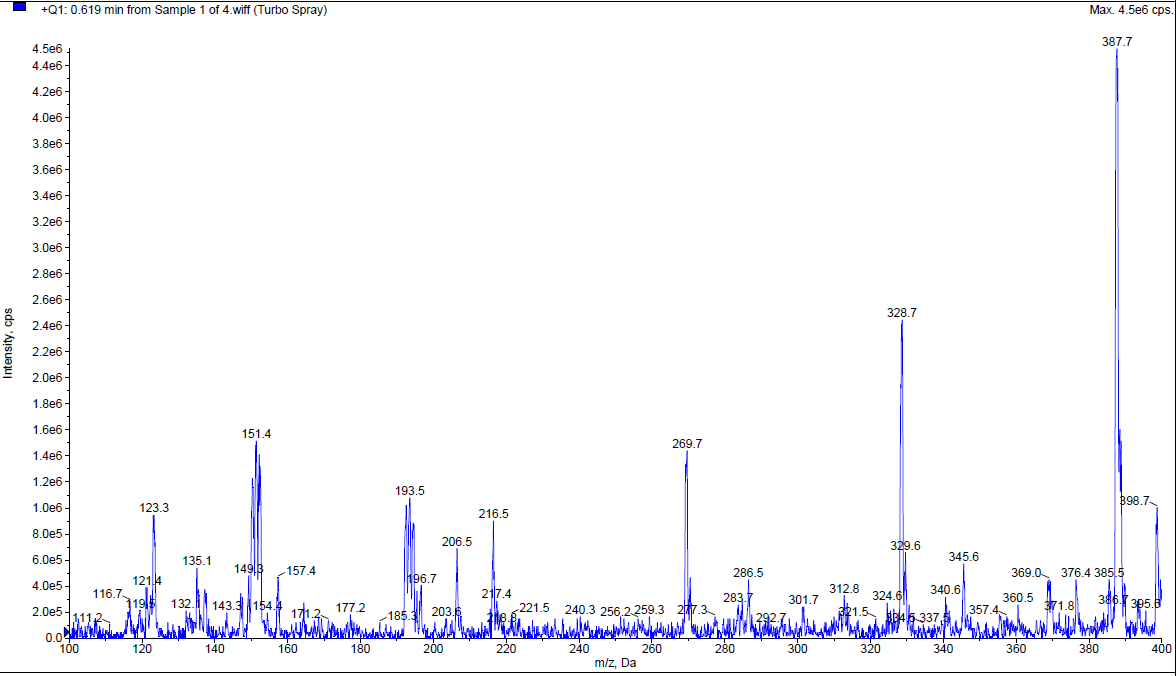


**Figure S13.** ESIMS spectrum of compound **3**


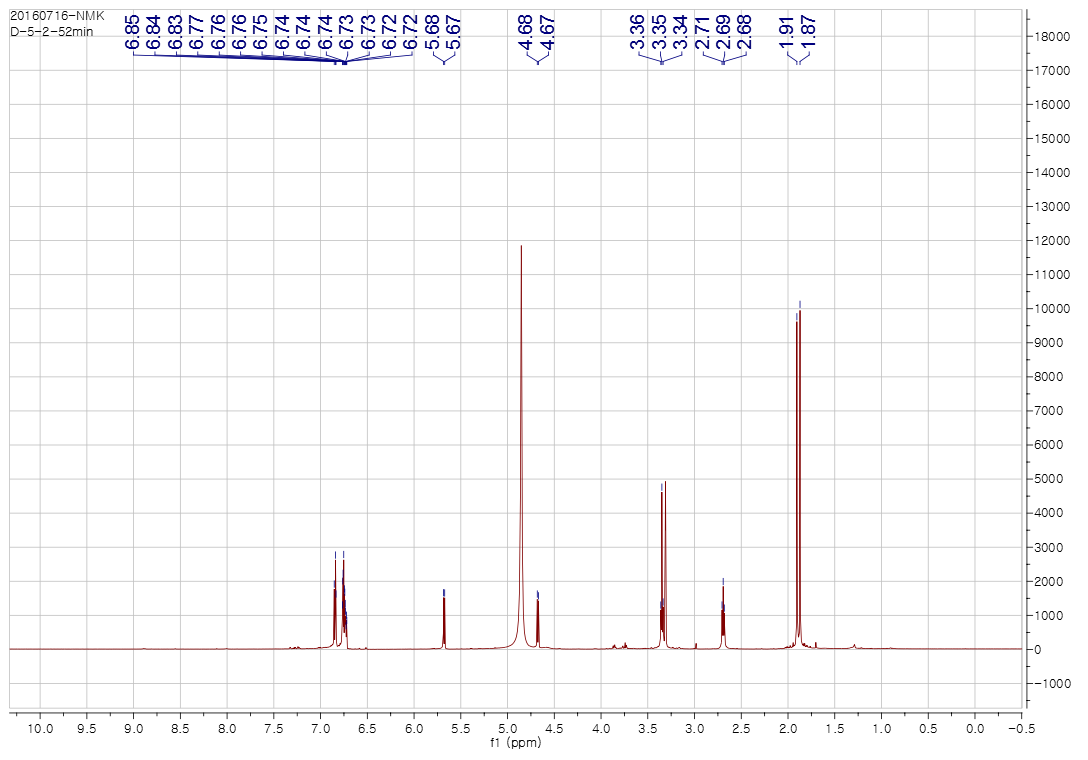


**Figure S14.** ^1^H NMR of compound **3** (600 MHz, methanol-*d_4_*)


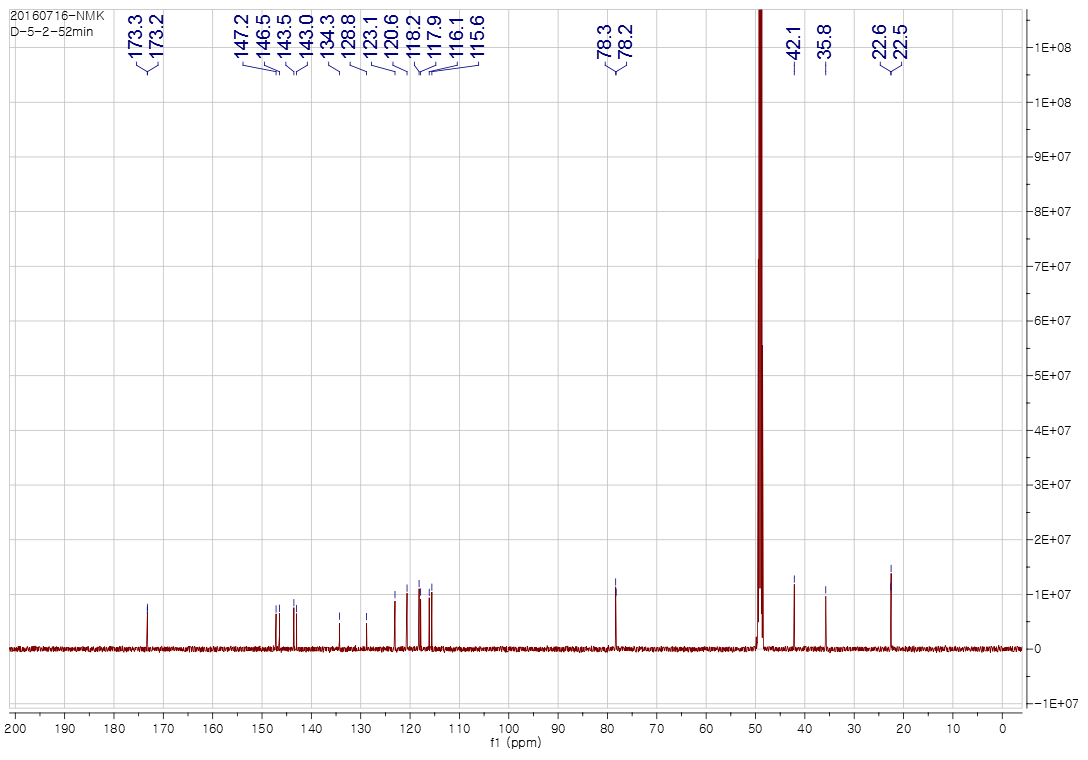


**Figure S15**. ^13^C NMR spectrum of compound **3** (150 MHz, methanol-*d_4_*)


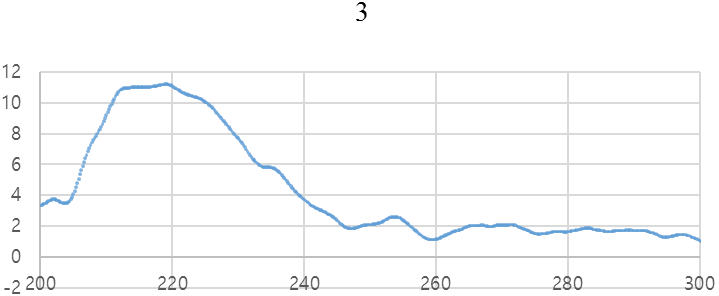


**Figure S16.** The CD spectrum of compound **3**


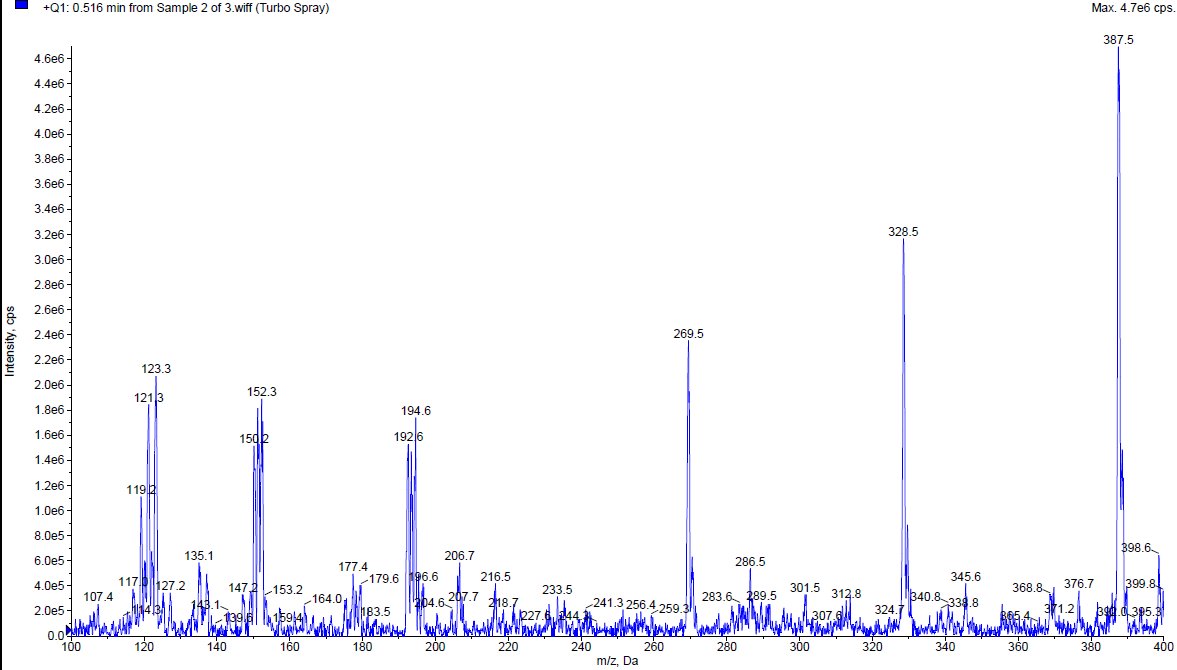


**Figure S17.** ESIMS spectrum of compound **4**


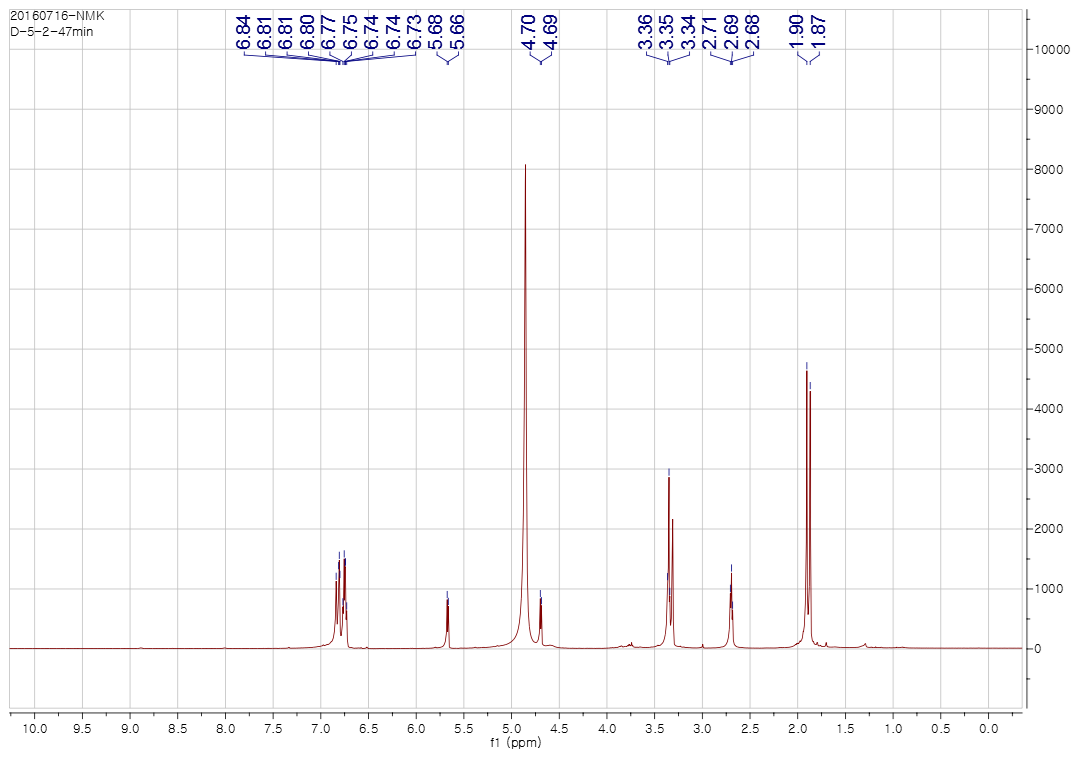


**Figure S18.** ^1^H NMR of compound **4** (600 MHz, methanol-*d_4_*)


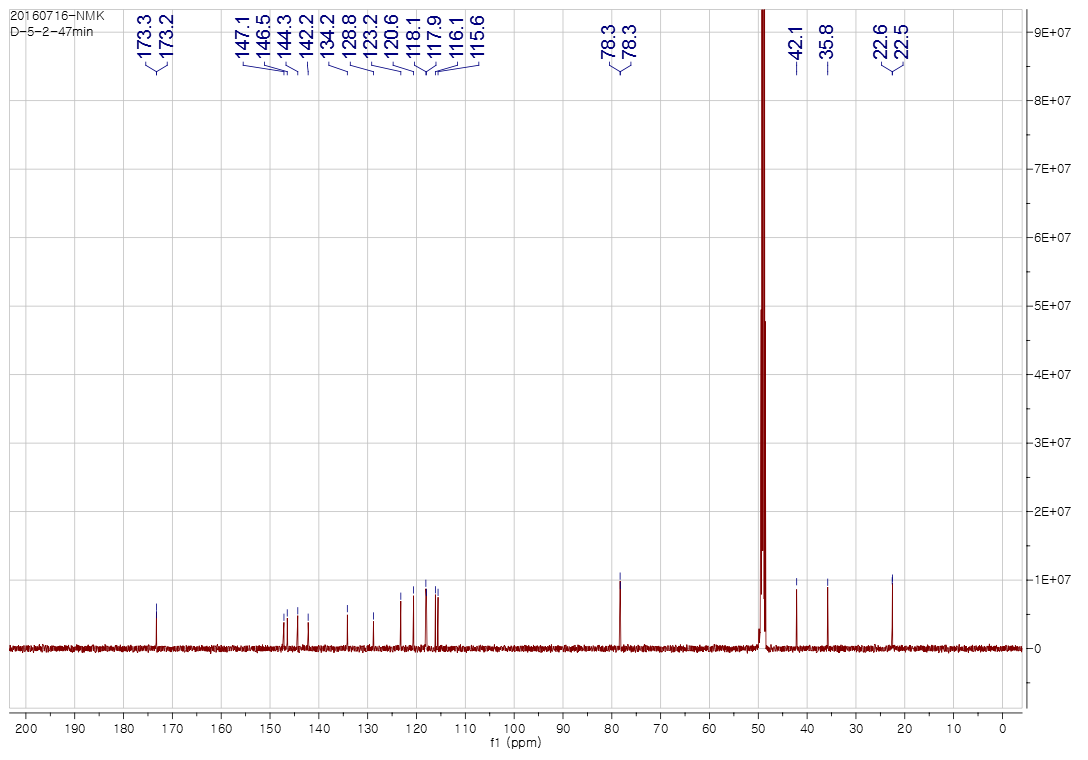


**Figure S19**. ^13^C NMR spectrum of compound **4** (150 MHz, methanol-*d_4_*)


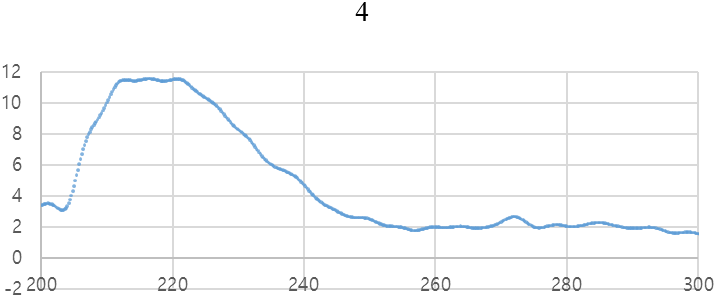


**Figure S20.** The CD spectrum of compound **4**


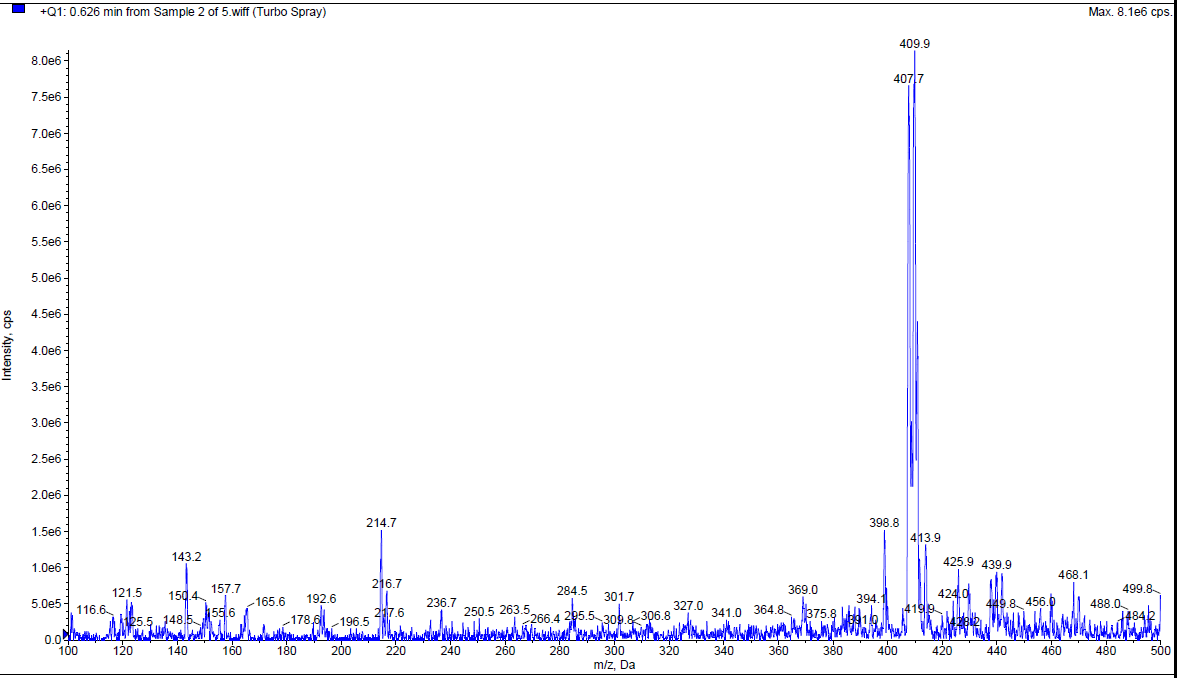


**Figure S21.** ESIMS spectrum of compound **5**


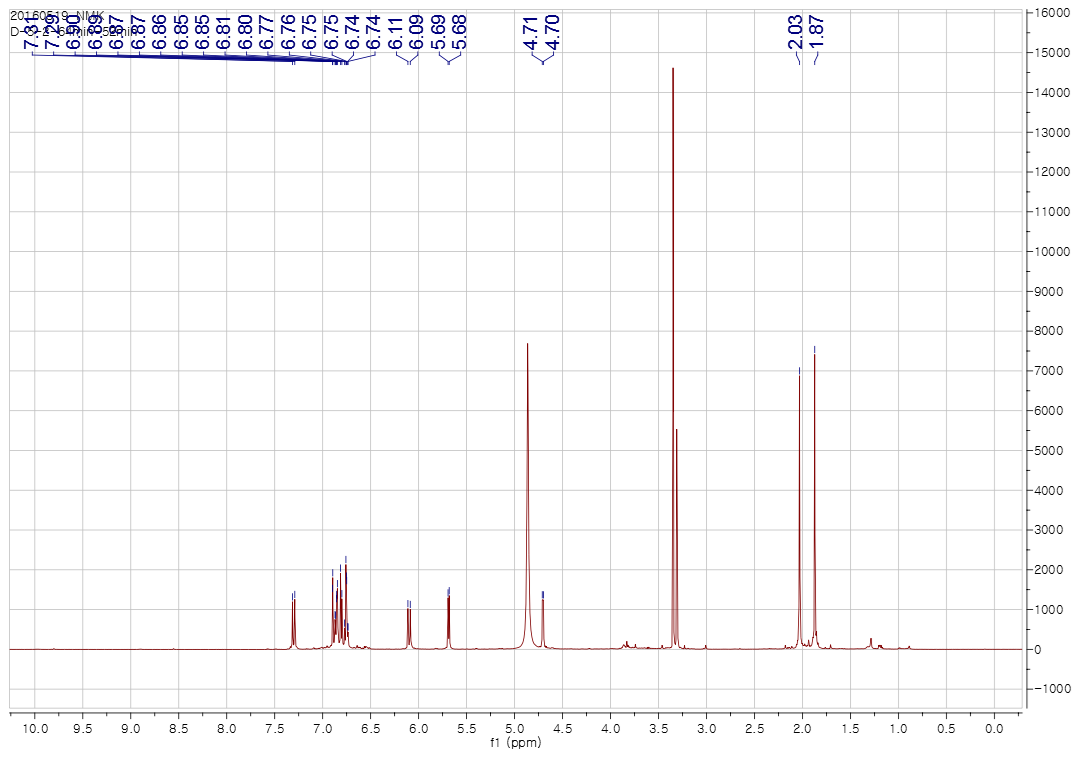


**Figure S22.** ^1^H NMR of compound **5** (600 MHz, methanol-*d_4_*)


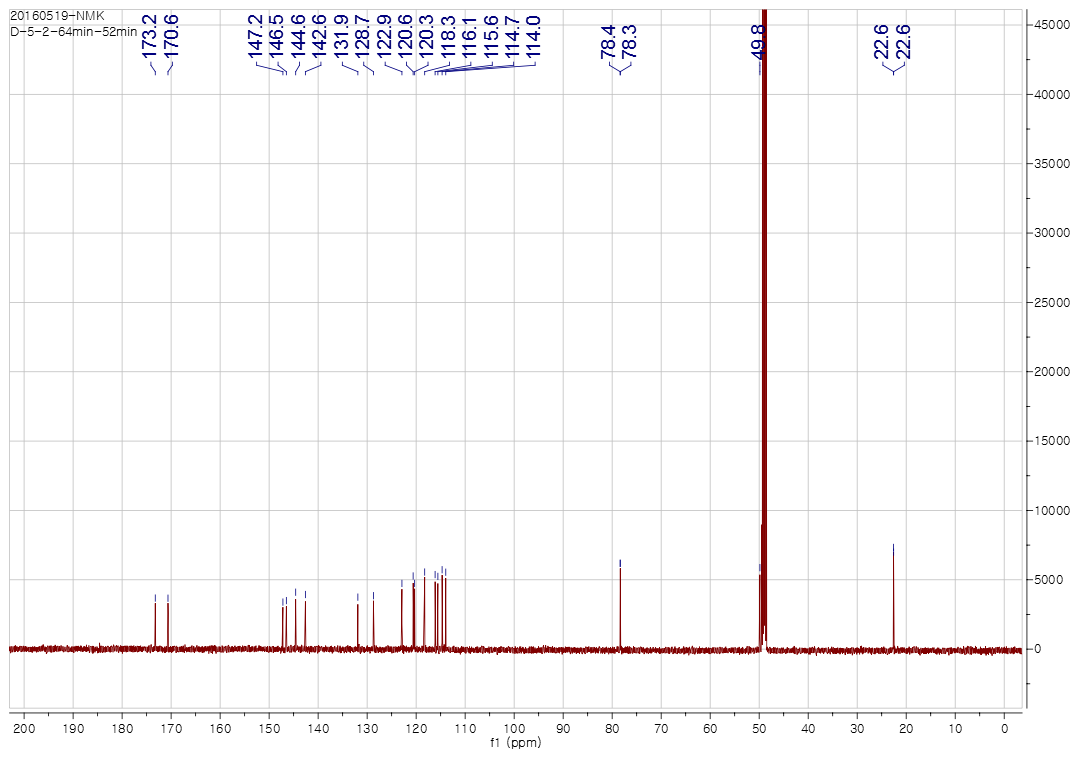


**Figure S23**. ^13^C NMR spectrum of compound **5** (150 MHz, methanol-*d_4_*)


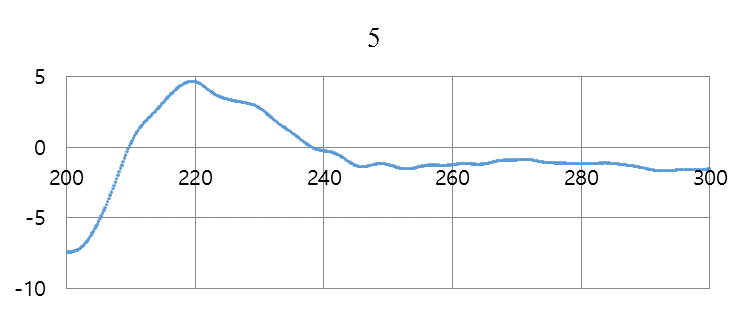


**Figure S24.** The CD spectrum of compound **5**

The associated chromatographic experimental details were elaborated in the *Extraction and Isolation* section.


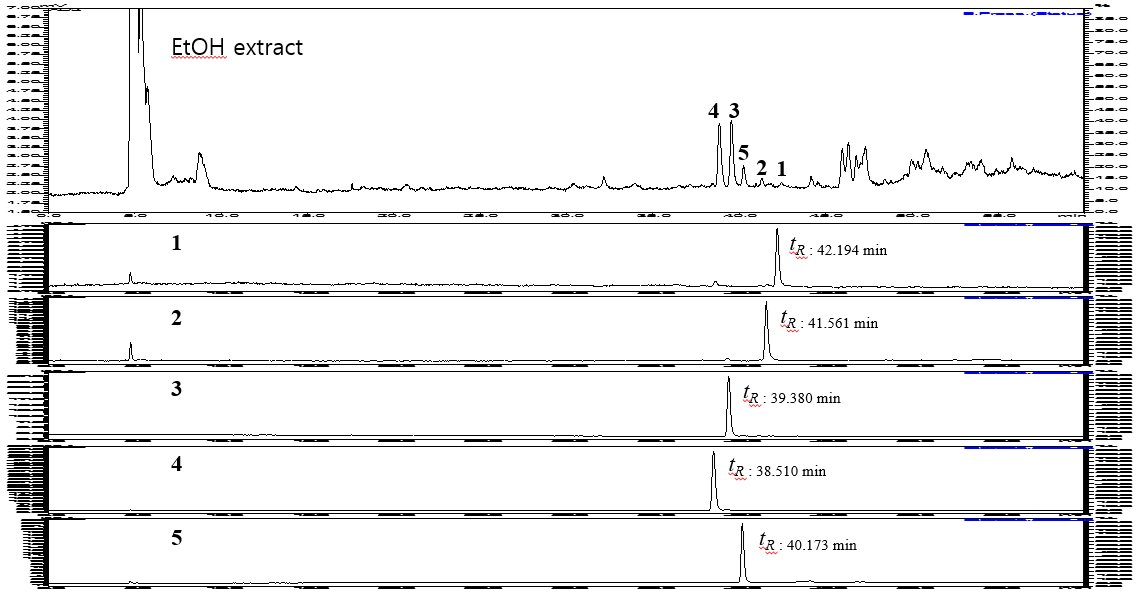


**Figure S25.** HPLC-ELSD profiling of compounds **1-5**
